# Swarm Autonomy: From Agent Functionalization to Machine Intelligence

**DOI:** 10.1002/adma.202312956

**Published:** 2024-05-02

**Authors:** Yibin Wang, Hui Chen, Leiming Xie, Jinbo Liu, Li Zhang, Jiangfan Yu

**Affiliations:** ^1^ School of Science and Engineering The Chinese University of Hong Kong Shenzhen 518172 China; ^2^ Shenzhen Institute of Artificial Intelligence and Robotics for Society Shenzhen 518172 China; ^3^ Department of Mechanical and Automation Engineering The Chinese University of Hong Kong Hong Kong 999077 China

**Keywords:** active matter, autonomy, machine intelligence, microrobots, swarm behavior

## Abstract

Swarm behaviors are common in nature, where individual organisms collaborate via perception, communication, and adaptation. Emulating these dynamics, large groups of active agents can self‐organize through localized interactions, giving rise to complex swarm behaviors, which exhibit potential for applications across various domains. This review presents a comprehensive summary and perspective of synthetic swarms, to bridge the gap between the microscale individual agents and potential applications of synthetic swarms. It is begun by examining active agents, the fundamental units of synthetic swarms, to understand the origins of their motility and functionality in the presence of external stimuli. Then inter‐agent communications and agent‐environment communications that contribute to the swarm generation are summarized. Furthermore, the swarm behaviors reported to date and the emergence of machine intelligence within these behaviors are reviewed. Eventually, the applications enabled by distinct synthetic swarms are summarized. By discussing the emergent machine intelligence in swarm behaviors, insights are offered into the design and deployment of autonomous synthetic swarms for real‐world applications.

## Graphical abstract

Swarm behaviors observed in nature have inspired the development of synthetic swarms. This review covers key aspects of synthetic swarms, including features of individual agents, external stimuli, swarm behaviors, and applications, examines underlying mechanisms of swarm generation and the emergence of machine intelligence, and envisions the realization of swarm autonomy.

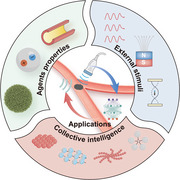

## 1 Introduction

In nature, swarm behaviors emerge through the self‐organization of large flocks of living organisms based on localized communication and decentralized decision‐making. These swarms perform functions beyond the capabilities of individual organisms leveraging the synchronization among a large number of individual units. Examples include the synchronization of light production in fireflies, the complex construction activities of bees and ants, and the pathogenic responses of bacteria.^[^ ^1^, ^2^, ^3^ ^]^ In the past few decades, considerable progress has been made in understanding and emulating the complex behaviors exhibited by natural swarms and translating these insights into the design of synthetic swarms. Here, synthetic swarms are defined as collectives composed of synthetic self‐propelling active agents, which can utilize the energy captured from external environments to generate motion. Swarm behaviors are used to describe the emergent collective behaviors of synthetic swarms. These swarm behaviors are predominantly governed by two factors: the motility of individual agents in the presence of external stimuli, and their communication with peer agents and the environment. The first factor involves various types of stimuli that agents can sense and the range of motility they can exhibit. The second factor involves the adaptation of agents when they interact with other agents and the environment. The combination of these two factors contributes to various swarm behaviors. These behaviors could enable extensive applications like material synthesis, micro‐nano manipulation, and biomedicine.

In this review, we start by offering an overview of the physical and chemical properties of individual active agents, revealing the origin of their motility and the functionalities they exhibit. Subsequently, we investigate the underlying mechanisms of swarm generation, focusing on the coordination between active agents that give rise to spontaneous order. The existing swarm behaviors are also summarized and their relationship with the features of agents and the types of external stimuli are discussed. The machine intelligence of synthetic swarms is categorized and discussed. Finally, the applications enabled by synthetic swarms are reviewed.

## 2 Features of Individual Active Agents

### 2.1 Motility of Active Agents

Active agents can be energized by external stimuli with various mechanisms. The motility of active agents can be categorized into random motion, short‐range directed motion, and long‐range directed motion. Short‐range directed motion is a phenomenon where active agents exhibit temporary directionality within a short time scale *t* (*t* ≤ 1/*D*_r_, where *D*_r_ refers to the rotational Brownian diffusion coefficient). Beyond *t*, their motion appears random due to the rotational diffusion. Long‐range directed motion is a phenomenon where active agents can maintain their direction over extended length and time scale (*t* > 1/*D_r_*), typically achieved by applying directed stimuli. The emergence of these motility types is influenced by both the structure attributes of the agents (isotropic or anisotropic) and the forms of the external stimuli (undirected or directed). In this section, we discuss the propelling mechanisms of diverse active agents.

#### 2.1.1 Propelling Isotropic Agents with Undirected Stimuli

**Figure 1:**
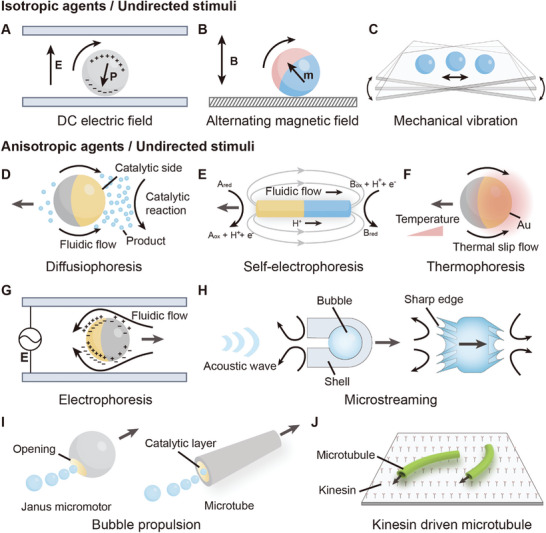
Schematic illustration of propulsion mechanisms for active agents based on undirected stimuli. A) Quincke rotation of an insulating microsphere under a direct current (DC) electric field. B) Random rotation of a magnetic microsphere under a uniaxial alternating magnetic field. C) Random oscillation of a microsphere on a vibrating plate. D) Diffusiophoresis of a catalytic Janus particle in the presence of chemical fuel. The yellow hemisphere indicates the catalytic side. E) Self‐electrophoresis of a catalytic Janus nanorod in the presence of chemical fuel. Gray arrows indicate the electric field. F) Self‐thermophoresis of an Au‐silica Janus particle. The yellow hemisphere indicates the Au side. G) Electrophoresis of a metal‐dielectric Janus particle under an alternating current (AC) electric field. The yellow hemisphere indicates the metal side. H) Microstreaming propulsion of acoustic microrobots in traveling acoustic waves. I) Bubble propulsion of a reactive Janus particle and a catalytic hollow tube. J) ATP‐energized propulsion of microtubules on a kinesin‐grafted substrate.

Undirected stimuli are external stimuli that do not exert directional torque or force to propel active agents in predefined directions. When subjected to such stimuli, isotropic active agents typically exhibit random motion. An example of self‐propelled active agents exhibiting random motion is the Quincke roller energized by the electric field.^[^ ^4^, ^5^, ^6^ ^]^ Subjected to a uniform direct current (DC) electric field, insulating spheres in conducting fluid experience charge accumulation, due to the electrical conductivity difference between the spheres and the medium. This leads to electric polarization. However, this charge distribution is inherently unstable and susceptible to infinitesimal fluctuations. Once the spontaneous symmetry breaking of the charge distribution occurs, it leads to electrostatic torque, causing the sphere to rotate in a random direction (**Figure** **1**A). Another example is ferromagnetic agents energized by the uniaxial alternating magnetic field (Figure 1B).^[^ ^7^ ^]^ The phase difference between the magnetization direction of the ferromagnetic roller and the external magnetic field results in a net torque, causing random rotational motion, either clockwise or counterclockwise. Since the field direction is orthogonal to the moving plane of the roller, the moving direction of the roller is random. Apart from the rollers that interact with the external field through induced polarization, passive particles can be energized through direct mechanical vibration. Utilizing a centrally actuated vibrating plate, such as a Chladni plate, passive particles can be actuated to perform random motion (Figure 1C).^[^ ^8^ ^]^

#### 2.1.2 Propelling Anisotropic Agents with Undirected Stimuli

Anisotropic active agents, when subjected to undirected stimuli, exhibit short‐range directed motion, intrinsically guided by their asymmetrical structure. However, achieving long‐range directed motion remains a challenge, due to the disturbance like rotational Brownian diffusion. This section overviews the actuation mechanisms of anisotropic active agents under undirected stimuli.

##### Diffusiophoresis

Janus particles, synthetic materials with two distinct hemispheres, are often propelled in chemical fuels via diffusiophoresis.^[^ ^9^, ^10^, ^11^, ^12^ ^]^ Here, the catalytic reaction on the active side of the Janus particle leads to a high local concentration of products, creating a concentration gradient. This concentration gradient induces osmophoresis, a process where water flows from the inert side with a low product concentration to the active side with a high product concentration, propelling the Janus particle toward its inert side (Figure 1D). Generally, the diffusiophoresis is dominated by the phoretic flow, especially when the products of the catalytic reaction are nonelectrolyte. The velocity of the propelled particle can be expressed as:^[^ ^13^ ^]^

(1) $$U=KL\frac{kT}{\eta }\nabla C$$

where U is the velocity of the particle, *K* is the Gibbs absorption length, *L* is the length of the particle‐solute interaction, *k* is the Boltzmann constant, *T* is the temperature, *η* is the viscosity, and *C* is the solute concentration.

##### Self‐Electrophoresis

Self‐electrophoresis is commonly observed in bimetallic catalytic rods, which create self‐generated electric fields and charged surfaces in chemical fuels.^[^ ^14^, ^15^, ^16^, ^17^, ^18^, ^19^, ^20^ ^]^ In contrast to the Janus particle with one active hemisphere and one inert hemisphere, both ends of the bimetallic nanorod are active, acting as catalysts for redox reactions. In a typical self‐electrophoresis process, redox reactions occur at opposite ends of the nanorod. Reductants are oxidized on one end of the nanorod (anode), releasing electrons and protons. At the opposite end (cathode), oxidants are reduced, consuming protons and electrons (Figure 1E). The resultant proton imbalance creates an ion gradient induced electric field, directing from the anode to the cathode. The mobile charges and the surrounding fluid near the nanorod migrate in response to the local electric field, propelling the nanorod in the opposite direction of the fluidic flow. A typical example of self‐electrophoresis involves Pt/Au colloidal rods self‐propelling in the presence of aqueous H_2_O_2_.^[^ ^18^ ^]^ These nanorods perform unidirectional motion in a short time scale of 1/*D_r_*, where *D_r_* refers to the rotational Brownian diffusion coefficient. While out of the time scale, the motion appears random due to rotational diffusion.

##### Thermophoresis

Thermophoresis describes the migration of colloidal particles or large molecules in fluid driven by temperature gradient.^[^ ^21^ ^]^ The self‐thermophoresis of Au‐silica Janus particles propelled by laser‐induced local temperature gradient has been investigated.^[^ ^22^ ^]^ The temperature gradient is generated by the laser absorption and the subsequent thermal effect on the Au side. Consequently, a thermal slip flow emerges, whose direction is influenced by the Soret coefficient (S_T_) of the Janus particle. When S_T_ > 0, the slip flow is directed from the cold region (silica side) to the hot region (Au side), propelling the Janus particle from the Au side to the silica side (Figure 1F).

##### Induced‐Charge Electrophoresis

Except for chemical fuels, Janus particles can also be energized by the external electric field. For example, the Janus particle composed of a metal hemisphere and a SiO_2_ hemisphere can be actuated in an alternating current (AC) electric field (Figure 1G).^[^ ^23^, ^24^ ^]^ In the electric field, the metal side of the Janus particle is more polarized than the SiO_2_ side. The different polarization of the two hemispheres creates an uneven charge distribution, resulting in a local electric field and ionic flow. Since the ionic flow moves from the less polarized SiO_2_ side to the more polarized metal side, the Janus particle is propelled toward the SiO_2_ side. This phenomenon is known as induced‐charge electrophoresis (ICEP).

##### Traveling Acoustic Wave

Traveling acoustic wave serves as an undirected stimulus in the fluidic environment. The propagation of sound represents the flow of energy. When the propagation is interrupted by an object, ultrasound exerts acoustic radiation pressure on the object which induces oscillation. Acoustic motors usually gain motility through oscillation‐induced microstreaming. There are two primary types of oscillation‐induced propulsion mechanisms: bubble oscillation and sharp‐edge oscillation (Figure 1H). Bubble oscillation induced propulsion leverages the expansion and contraction of an entrapped air bubble within the cavity of an acoustic motor under the traveling acoustic wave in high frequency. As the bubble oscillates, liquid flows in and out of the cavity of the acoustic motor, generating a quasistatic microstream with net momentum, thereby propelling the acoustic motor.^[^ ^25^, ^26^, ^27^ ^]^ Sharp‐edge propulsion, on the other hand, utilizes the interaction between sharp structures on active agents and acoustic waves. When acoustic motors with sharp structures are exposed to traveling acoustic waves, counter‐rotating vortices are generated on both sides of sharp edges, inducing net flux flows away from them, which provide propulsion for acoustic motors.^[^ ^28^, ^29^ ^]^ Advanced acoustic motors, which incorporate efficient hydrodynamic structures and ciliary bands, have been developed to enhance propulsion efficiency.^[^ ^30^ ^]^ Notably, the propulsion direction of these motors is determined by their structures, rather than the direction of the traveling acoustic waves, as the generation of microstream relies solely on the anisotropic structure of the acoustic motors.

##### Bubble Propulsion

Bubble propulsion is generally observed in Janus particles or microtubes.^[^ ^30^, ^31^, ^32^, ^33^ ^]^ For example, microspheres of active metal (for example, Mg) or catalytic materials (for example, Pt, TiO_2_) can be partially functionalized with an inert protective layer, which leads to chemical reactions on one side. The resulting gas generation on the active side of the Janus particle provides net propulsion force (Figure 1I).^[^ ^31^, ^34^, ^35^ ^]^ A typical tubular structure propelled by bubble propulsion comprises an inert shell and a catalytically active platinum layer on the interior surface (Figure 1I). In the presence of chemical fuels, catalytic reaction generates gas within the tubular structure. These tubular agents are usually designed with asymmetrical structures, facilitating gas release from one end and propelling these motors in the opposite direction.

##### Molecular Motors

Molecular motors are molecular systems that convert chemical energy into mechanical work, which play crucial roles in various cellular processes. These systems are typically composed of biomolecular motors and their corresponding cytoskeletal filaments. Key examples include myosin‐actin, dynein‐microtubule (dynein‐MT), and kinesin‐microtubule (kinesin‐MT) systems.^[^ ^36^, ^37^ ^]^ Taking the kinesin‐MT system as an example, leveraging the hydrolysis of ATP, microtubules (MT) can move smoothly on the kinesin‐grafted glass substrate (Figure 1J). The motion of microtubules does not have preferred directionality. Additionally, microtubules can be easily integrated with proteins, crosslinkers, or DNAs to regulate their mutual interactions and further lead to the emergence of complex swarm behaviors.

#### 2.1.3 Propelling Active Agents with Directed Stimuli

Directed stimuli can be used to propel active agents in a specific direction. There are two primary ways to pose directed stimuli to active agents. One strategy involves employing physical fields that exert the desired torque and force directly on the agents, such as magnetic field, electric field, and acoustic field. Another method involves inducing asymmetrical chemical reactions around catalytic active agents with asymmetrical illumination or chemical fuel gradient. These directed stimuli enable active agents to maintain long‐range directionality even in the presence of disturbances. This section overviews the directed stimuli that propel active agents and investigates the underlying mechanisms.

##### Magnetic Field

**Figure 2:**
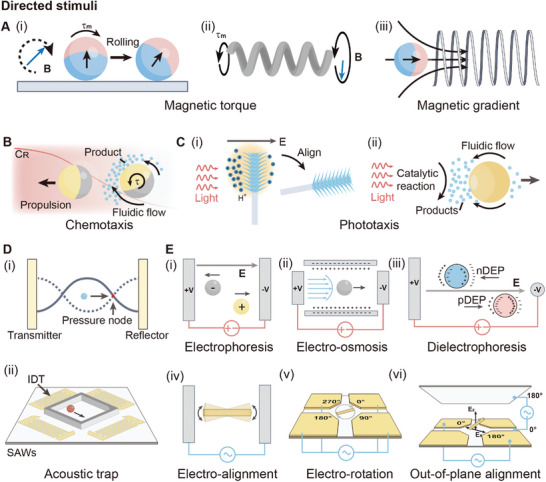
Schematic illustration of propulsion mechanism for active agents based on directed stimuli. A) Active agents propelled by magnetic fields. i) Unidirectional rotation of a magnetic roller in a rotating magnetic field. ii) Helical propulsion of a magnetic helix in a rotating magnetic field. iii) Translational motion of a magnetic particle in the magnetic field generated by a current‐carrying solenoid. B) Self‐alignment and chemotaxis of a catalytic Janus particle in the presence of chemical fuel with a concentration gradient. The red curve indicates that the chemical concentration decrease from the left to the right. The yellow hemisphere indicates the catalytic side. C) Phototaxis of active agents. i) Phototaxis of a Janus nanotree in the presence of asymmetrical light. Reproduced with permission.^[^ ^38^ ^]^ Copyright 2016, Springer Nature. ii) Phototaxis of an isotropic semiconductor micromotor. D) Agents propelled with i) bulk acoustic waves, and ii) surface acoustic waves. E) Propulsion of active agents based on electrokinetic mechanisms, including i) electrophoresis, ii) electro‐osmosis, iii) dielectrophoresis, iv) electro‐alignment, (v) electro‐rotation, and vi) out‐of‐plane alignment.

Magnetic active agents refer to agents incorporating magnetic components that can be actuated by external magnetic fields.^[^ ^39^ ^]^ Based on their hysteresis properties, these magnetic agents can be divided into two categories: ferromagnetic materials with high magnetic hysteresis, and paramagnetic materials with low magnetic hysteresis. Ferromagnetic materials, once magnetized to saturation, become stable and permanent sources of magnetic field. In contrast, the magnetic moment of paramagnetic materials is induced by an external magnetic field and vanishes when the field is removed.^[^ ^40^ ^]^ The propulsion of these agents relies on magnetic torque and magnetic force. Magnetic torque arises from the difference between the magnetization direction of the magnetic agents and the direction of the external magnetic field, which can be expressed as:

(2) τ=m×B

where **m** is the magnetic moment of the active agents, **B** is the external magnetic field. Leveraging magnetic torque, the tumbling of magnetic rollers and the helical propulsion of magnetic helices can be realized in rotating magnetic fields (**Figure** **2**A).^[^ ^41^, ^42^ ^]^ The rolling direction of the magnetic roller aligns with the plane of rotating magnetic field, while the propulsion direction of the magnetic helix is perpendicular to the plane. Magnetic force is only present in the presence of magnetic fields gradient. The magnetic force exerted on a magnetic agent in a magnetic field can be expressed as:

(3) F=(m·∇)B

In the magnetic field, magnetic agents experience both magnetic torque and force. The torque aligns their magnetic moment with the field, while the force attracts the aligned agents toward the field source.

##### Chemical Fuel Gradient

Chemotaxis is essential for microorganisms in seeking nutrients and avoiding toxins.^[^ ^43^ ^]^ This swarm behavior, characterized by movement toward (positive chemotaxis) or away from (negative chemotaxis) chemical source, is not only observed in biological systems but also replicated in synthetic swarms.^[^ ^44^, ^45^, ^46^ ^]^ When exposed to uniform chemical fuels, catalytic active agents perform undirected locomotion through mechanisms like diffusiophoresis or self‐electrophoresis induced by chemical reactions. The phoretic flow rate depends on the local catalytic reaction rate, which is proportional to the local fuel concentration. The presence of a global concentration gradient leads to varying reaction rates across different sites of an active agent, resulting in uneven distribution of products. The nonuniform distribution of product concentration along the particle gives rise to a phoretic flow that aligns the particle's catalytic side toward high concentration region (Figure 2B). The reoriented agent can then propel toward its catalytic side, exhibiting positive chemotaxis. By adjusting the phoretic mobility on different sides of the Janus particle, negative chemotaxis can also be realized.^[^ ^38^ ^]^

##### Light

Phototaxis, a phenomenon observed in photosynthetic microorganisms, involves movement toward or away from light sources for energy acquisition or predator avoidance. This behavior has been replicated in photoactive agents. Unlike chemotaxis, which relies on the unbalanced slip flow induced by the concentration gradient of chemical fuels, optical signals provide a way to guide active agents remotely. One typical example of such agents is the Janus TiO_2_/Si nanotree.^[^ ^38^, ^47^ ^]^ Its motility originates from photochemical reaction induced electrophoresis, with the TiO_2_ head and Si tail acting as photoanode and photocathode, respectively. Under global illumination, a photoelectrochemical reaction occurs, leading to proton generation at the TiO_2_ side and proton consumption at the Si side. The reaction results in a proton imbalance, which builds up an electric field, leading to self‐electrophoresis. When the Janus nanotree is illuminated from one side, the short light absorption length of the TiO_2_ nanowire array results in higher photon absorption and proton generation on the illuminated side. This leads to the generation of an electric field parallel to the direction of the incident light, pointing from the illuminated side to the shaded side (Figure 2C). Because the TiO_2_ head is positively charged, the electric field rotates the nanotree and pushes it away from the light source. For isotropic photoactive agents, phototaxis can be realized by leveraging a local chemical concentration gradient induced by asymmetrical chemical reactions.^[^ ^48^, ^49^ ^]^ When a beam of light illuminates the photoactive semiconductor, the light intensity attenuates exponentially with the increase of penetration depth. As a result, the chemical reaction rate on the illuminated side is higher than that on the shaded side, leading to an uneven distribution of products. This uneven distribution creates a local electric field that propels the agent to the shaded side, demonstrating a negative phototaxis (Figure 2C).^[^ ^48^ ^]^ The isotropic structure ensures that the propelling direction of the agent aligns consistently with the direction of light, unaffected by rotational Brownian motion.

##### Standing Acoustic Wave

Acoustic waves can be employed to actuate active agents through their interaction with solids, liquids, and gases.^[^ ^50^, ^51^, ^52^ ^]^ As previously mentioned, traveling acoustic waves can be considered as undirected stimuli. In contrast, within standing acoustic waves, active agents experience directional acoustic radiation force. The standing acoustic waves can be categorized into bulk acoustic waves (BAWs) and surface acoustic waves (SAWs).^[^ ^52^ ^]^ A typical BAWs resonator consists of a piezoelectric transducer and a reflector, or two opposing transducers with the same frequency. Standing acoustic waves in such setups are formed by the superposition of two traveling waves of the same frequency but in opposite direction (Figure 2D(i)). This also leads to the formation of nodes (points of zero amplitude) and antinodes (points of maximum amplitude). Within a standing acoustic wave, the active agent is propelled to nodes by acoustic radiation force. The force experienced by an active agent in the acoustic field can be expressed as:^[^ ^50^ ^]^

(4) $$F_{\mathrm{AX}}=4\pi R^{3}Ek\sin\left(2kx\right)\Phi$$

(5) $$k=\frac{2\pi f}{c_{0}}$$

(6) $$\Phi =\frac{\rho_{p}+\left(2/3\right)\left(\rho_{p}-\rho_{0}\right)}{2\rho_{p}+\rho_{0}}-\frac{1}{3}\frac{\rho_{0}{c_{0}}^{2}}{\rho_{p}{c_{p}}^{2}}$$

where *F_AX_* is the primary axial radiation force, *E* is the acoustic energy density, *R* is the particle radius, *x* is the particle position in the wave propagation direction, *ρ*_*p*_ and *ρ*_0_ are densities of particle and fluid, respectively, *c_p_* and *c*_0_ are speed of sound in solid and fluid, respectively, *f* is the frequency, and Φ is the acoustic contrast factor. By changing the frequency of the acoustic wave, the positions of pressure nodes and antinodes can be further adjusted to generate the desired pressure distribution.

Surface acoustic waves are generally generated by interdigital transducers (IDTs) patterned on a piezoelectric substrate.^[^ ^53^ ^]^ By applying an alternating voltage on the IDTs, a periodic strain field is generated, thereby generating a surface acoustic wave propagating along the IDTs. Orthogonally arranged pairs of IDTs create arrays of potential wells at the intersection of two standing waves, acting as trapping sites for active agents (Figure 2D(ii)).^[^ ^54^, ^55^, ^56^, ^57^, ^58^ ^]^ The acoustic radiation force acting on these agents is determined by the gradient of the potential field, which depends on factors such as the size and density of the active agents, properties of the surrounding fluids, frequency of the SAWs, and power of the IDTs. By implementing phase shift and amplitude modulation of SAWs, real‐time in‐plane manipulation and out‐of‐plane lifting of active agents can be achieved.^[^ ^53^ ^]^ Furthermore, advanced IDT designs, like IDTs arrays with spiral configurations, further enable the dynamic reshaping of SAW fields to programmed configurations, thereby achieving more complicated particle manipulations.^[^ ^59^ ^]^

##### Electric Field

The electric field offers various ways to energize active agents through electrokinetic mechanisms like electrophoresis, electro‐osmosis, dielectrophoresis, electro‐alignment, and electro‐rotation. Electrophoresis describes the translational motion of charged agents in static electric fields (Figure 2E(i)). The force originates from the Coulombic force acting on the charges.^[^ ^60^, ^61^ ^]^ The electrophoretic motion is governed by the Helmholtz–Smoluchowsky equation, which relates the dielectrophoretic mobility of active agents to their zeta potential.^[^ ^62^ ^]^ The Helmholtz–Smoluchowsky equation is expressed as:

(7) $$\mu_{e}=\frac{\varepsilon_{r}\varepsilon_{0}\xi_{a}}{\eta }$$

where ɛ_*r*_ is the dielectric constant of the dispersion medium, ɛ_0_ is the permittivity of free space, *η* is the dynamic viscosity of the medium, and *ξ*_*a*_ is the zeta‐potential of the agent. In an external electric field *E*, the velocity of the charged particle can be expressed as *v* = *E*µ_*e*_.

Electro‐osmosis occurs when an electric field is applied orthogonal to a channel filled with electrolytes.^[^ ^63^, ^64^, ^65^, ^66^ ^]^ This mechanism involves the movement of ions in the electrical double layer (EDL) formed on charged channel surfaces. Upon applying an electric field to the electrolyte, the charge in the EDL experiences Coulombic force and thus generates a net flow in the channel. Agents in the medium can thus be propelled by the electro‐osmosis flow (Figure 2E(ii)). The velocity of the electro‐osmosis flow caused by an electric field with a strength of *E* can be expressed as:^[^ ^62^ ^]^

(8) $$u=-\frac{\varepsilon_{r}\varepsilon_{0}\xi_{s}}{\eta }E$$

where ɛ_*r*_ is the dielectric constant of the dispersion medium, ɛ_0_ is the permittivity of the free space, *η* is the dynamic viscosity of the medium, and *ξ*_*s*_ is the zeta‐potential of the substrate.

Dielectrophoresis is commonly observed in an inhomogeneous electric field, such as the electric field generated by a wire‐plate electrode arrangement.^[^ ^67^ ^]^ Unlike the previously mentioned electrophoresis which primarily depends on the charge carried by the agents, dielectrophoresis arises from the polarization of agents. When subjected to an external electric field, the active agents polarize, resulting in charge separation. Charges of opposing signs but equal magnitude accumulate on the opposite sides of the polarized particle. Moreover, the surrounding medium also polarizes, with charges of opposite sign accumulating at the liquid side of the liquid–solid interface, a phenomenon known as the Maxwell–Wagner interfacial polarization (Figure 2E(iii)). The extent of polarization depends on the permittivity of the materials. When the agent is better polarized than the medium, more charge accumulates on the agent, resulting in a dipole moment (from the negative charge to the positive charge) along the electric field direction. The resulting dielectrophoresis is termed positive dielectrophoresis (pDEP), in which the agent moves along the electric field. Conversely, if the agent is less polarized, it experiences negative dielectrophoresis (nDEP) and moves against the direction of the electric field. The dielectrophoretic force experienced by a spherical particle in an electrical field can be expressed as:^[^ ^67^, ^68^ ^]^

(9) $${F_{DE}}_{P}=\pi \varepsilon_{m}\varepsilon_{0}a^{3}Re\left[K_{CM}\right]\nabla {\left|E\right|}^{2}$$

where ɛ_*m*_ and ɛ_0_ are the relative permittivity of the medium and the free space, respectively. *K_CM_* is the particle's Clausius–Mossotti factor, whose real part gives the relative polarizability of the agent in the medium.

Electro‐alignment is usually observed in longitudinal agents, where they align in an alternating electric field (AC E‐field) due to the interaction between their real‐part electric polarization and the electric field. For example, a silicone nanowire in an AC E‐field with frequencies ranging from kilohertz to megahertz aligns its long axis instantly with the field direction, driven by dipole‐induced torques (Figure 2E(iv)). The torque can be expressed as:^[^ ^69^ ^]^

(10) $$\tau_{A}=\frac{E^{2}}{2}Re\left[\alpha_{a}-\alpha_{b}\right]\cos\theta \sin\theta$$

where *θ* is the angle between the electric field and the long axis of the particle, *α*_*a*_ and *α*_*b*_ are the complex polarizabilities along the long axis and the short axis of the agent, and Re is the Reynolds number.

Electro‐rotation of active agents can be achieved using a rotating electric field. The rotating electric field can be generated using quadruple microelectrodes with sequential phase shifts of 90° (Figure 2E(v)).^[^ ^70^, ^71^, ^72^ ^]^ The resulting torque on the polarized agents is given by:

(11) $$\tau_{R}=\frac{2\pi }{3}r^{2}l\varepsilon_{m}Im\left(K\right)E^{2}$$

where *r*, *l* are the radius and length of the nanowire, ɛ_*m*_ is the permittivity of the suspension medium, and Im(K) is the imaginary part of the Clausius–Mossotti factor *K* of the nanowire. By further implementing quadruple microelectrodes with a pair of vertically placed electrodes, precise transport, rotation, and alignment of the polarized agents in‐plane or out‐of‐plane can be realized, through the combination of multiple electrokinetic effects (Figure 2E(vi)). This enables the precise manipulation of objects at subcellular resolution.^[^ ^72^, ^73^, ^74^ ^]^

#### 2.1.4 Propulsion Mechanisms of Biohybrid Active Agents

Biohybrid active agents indicate active agents comprising both synthetic and biological components, such as bacteria‐driven microswimmers and magnetically powered neutrophils.^[^ ^75^, ^76^, ^77^ ^]^ Biological components of the biohybrid agents can provide propulsion forces and torques to generate translational and rotational motion, by utilizing the intrinsic motility of microorganisms. Microorganisms have evolved unique propulsion mechanisms to perform efficient locomotion in the low Reynolds number regime. For instance, flagellated bacteria rotate their flagella to generate thrust force,^[^ ^78^, ^79^ ^]^ eukaryotic cells swim with periodic flagellar motion,^[^ ^80^ ^]^ and microalgae generate propulsion with nonreciprocal strokes of their flagella.^[^ ^78^ ^]^

Microorganisms can exhibit autonomy and intelligence by responding to environmental signals, such as chemicals, temperature, and light, through tactic response. Applying and tuning external stimuli is one of the methods to control biohybrid active agents. Microalgae‐based biohybrid robots have been developed by modifying microalgae with polymer colloids through EDC/NHS reaction.^[^ ^81^ ^]^ These robots can be guided by visible light, leveraging the phototactic response of microalgae. By using other surface functionalization strategies, including noncovalent binding, covalent binding, cell penetration, and encapsulation, various microalgae‐based microrobots have been developed for different applications, such as drug delivery, wound healing, water purification, and cargo transportation.^[^ ^77^, ^82^, ^83^, ^84^, ^85^ ^]^ Biohybrid active agents propelled and controlled solely by the tactic response of bacteria are also developed.^[^ ^86^, ^87^ ^]^ The directional guidance of biohybrid active agents can also be achieved through externally controlling the synthetic components.^[^ ^88^, ^89^, ^90^ ^]^ Sperm cells can be trapped in magnetic microstructures, resulting in sperm‐based microrobots, where sperm provide propulsion force while the direction of the microrobot can be controlled by external magnetic fields.^[^ ^89^, ^90^ ^]^

### 2.2 Structure of Active Agents

**Figure 3:**
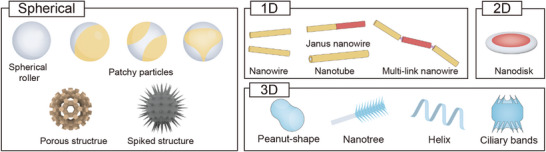
Active agents with different morphology. Active agents can be categorized based on their morphologies into spherical, 1D, 2D, and 3D structures.

Based on the morphology of active agents, they can be classified into spherical, 1D, 2D, and 3D structures (**Figure** **3**). Examples of spherical active agents include iron oxide nanoparticles and nickel nanoparticles energized by magnetic fields,^[^ ^7^, ^91^ ^]^ and polystyrene particles energized by electric fields.^[^ ^4^, ^92^ ^]^ By depositing materials with different compositions on the surfaces of spherical particles, patchy particles can be created. The most commonly used patchy particles are Janus particles featuring two distinct compositions on opposite sides. They usually demonstrate asymmetrical motility due to their structural anisotropy. Various Janus particles with distinct properties have been developed, such as magnetic Junus particles,^[^ ^42^, ^93^ ^]^ catalytic Janus particles,^[^ ^11^, ^12^ ^]^ and metal‐dielectric Janus particles.^[^ ^23^, ^94^ ^]^ By depositing different materials on two poles of spherical particles, triblock particles can be fabricated. Triblock particles can act as unique building blocks with site‐selective interparticle interactions, which contribute to complex swarm behaviors like programmed self‐assembly.^[^ ^95^, ^96^, ^97^ ^]^ Patchy particles with more complex patterns are also developed, which can perform nonlinear helical motion under an electric field, due to asymmetrical metal patches on the particle surface.^[^ ^98^ ^]^ Based on spherical particles, active agents with substructures, such as porous structures and spiked structures, are created. These structures are mainly designed for specific applications but not related to their motilities. Porous structures provide abundant sites for loading various cargos including drugs and therapeutic cells, thereby facilitating the biomedical application of active agents. Spiked structures offer anchoring sites for active agents, enabling greater tissue penetration, which contributes to the long‐time retention of functional active agents in the human body.^[^ ^99^, ^100^ ^]^ 1D active agents refer to nanowires, nanotubes, and their derivatives. The fabrication of 1D active agents usually involves templated deposition. Depending on the materials used, these agents can be energized by magnetic fields, electric fields, or chemical fuels.^[^ ^18^, ^73^, ^101^, ^102^ ^]^ Specifically designed active agents with 1D structures like multilink nanowires exhibit unique motility in low Reynolds number environments.^[^ ^103^ ^]^ For 2D active agents, microdisks with electrical response or magnetic response have been developed.^[^ ^104^, ^105^, ^106^ ^]^ Their flat shape facilitates the study of swarm behaviors at the fluid‐air interface.

Advancements in micro‐nanofabrication have also enabled the creation of active agents with complex 3D structures. For example, magnetic peanut‐shaped particles can be fabricated through hydrothermal synthesis,^[^ ^107^ ^]^ photoactive nanotrees can be fabricated with a sequential chemical deposition method, ^[^ ^38^ ^]^ and active agents with helical structures can be fabricated through glancing angle deposition (GLAD) or 3D printing.^[^ ^108^, ^109^, ^110^, ^111^, ^112^ ^]^ With two‐photon polymerization 3D printing, a wide variety of microscale agents with distinct 3D structures can be manufactured, for example, acoustic motors with cilia bands,^[^ ^29^ ^]^ and magnetic drug carriers with hierarchical porous structures.^[^ ^99^ ^]^

### 2.3 Functionalization of Active Agents

**Figure 4:**
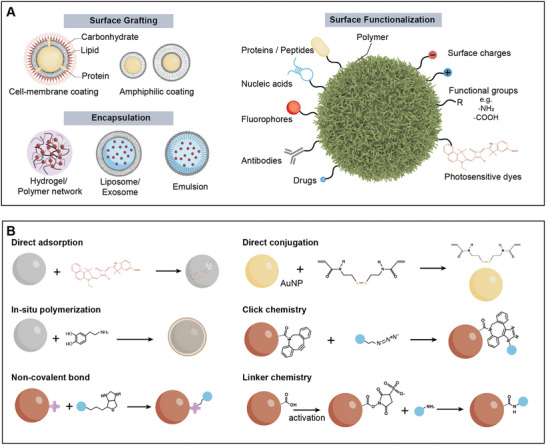
Functionalization of active agents. A) Design principles of functionalized active agents. These agents are generally functionalized by surface grafting, encapsulation, and surface functionalization. The functionalization mainly contributes to the responsive properties and functionalities of active agents. B) Approaches used for functionalizing active agents.

Functionalization of active agents plays a pivotal role in regulating their interparticle interactions and broadening their practical applications. In general, active agents can be functionalized in mainly three ways, surface grafting, encapsulation, and surface functionalization (**Figure** **4**). Surface grafting involves coating active agents with materials like cell membranes or amphiphiles, which act as camouflage. Such modifications are particularly relevant to biomedical applications, resulting in active agents with enhanced biocompatibility, immune evasion, and tumor targeting.^[^ ^75^, ^100^, ^113^ ^]^ Encapsulation involves embedding a group of agents within a single capsule, in the form of hydrogel/polymer networks,^[^ ^114^ ^]^ liposomes or exosomes,^[^ ^115^, ^116^ ^]^ and emulsion droplets.^[^ ^117^, ^118^ ^]^ Encapsulation of active agents not only aids in studying swarm behaviors in confined spaces but also facilitates the development of novel biomedicine carriers. By directly engineering the surface of active agents, various surface functionalizations can be accomplished for distinct purposes. This includes modifications of proteins, peptides, and antibodies for targeted recognition, DNA, surface charges, and functional groups for regulating interparticle interactions, fluorophores for imaging and labeling, drugs and nucleic acids for therapeutic applications, and photosensitive dyes for photoactivation. Such versatile functionalizations significantly enhance the capabilities of active agents in terms of mutual interactions, stimuli‐responsive properties, and application‐oriented functionalities, thus enriching their swarm behaviors and practical applications. Various approaches have been developed for surface functionalization, examples include direct adsorption of photosensitive dye on photoactive motors,^[^ ^38^, ^119^ ^]^ direct conjugation of thiol derivatives on gold nanoparticles through Au‐thiolate interaction,^[^ ^120^ ^]^ in situ polymerization of polydopamine on magnetic particles,^[^ ^121^ ^]^ click chemistry enabled nanoparticle modification on algae motors,^[^ ^83^, ^84^, ^85^ ^]^ noncovalent modification of drugs or antibodies with biotin‐streptavidin conjugation, and covalent functionalization via linker chemistry, such as EDC/NHS reaction.

## 3 Mechanisms for Swarm Generation

Swarm generation is achieved through individual active agents interacting with one another and their environment. Although the rules of interaction can be relatively simple, they lead to the emergence of complex collective motion through decentralized decision‐making. In this section, we discuss the underlying mechanisms that induce swarm generation, focusing on both inter‐agent interactions and agent‐environment interactions.

### 3.1 Inter‐Agent Interactions

Interactions described in colloidal science (e.g., electrostatic and hydrophobic interactions)^[^ ^122^, ^123^ ^]^ and time‐dependent nonequilibrium interactions induced by external stimuli (e.g., phoretic interaction and dynamic dipolar interactions) are considered inter‐agent interactions. These interactions lead to the alignment, attraction, or repulsion of neighboring self‐propelling agents, and eventually contribute to the swarm generation on a larger scale. This section reviews inter‐agent interactions induced by different mechanisms.

#### 3.1.1 Collision‐Induced Alignment

**Figure 5:**
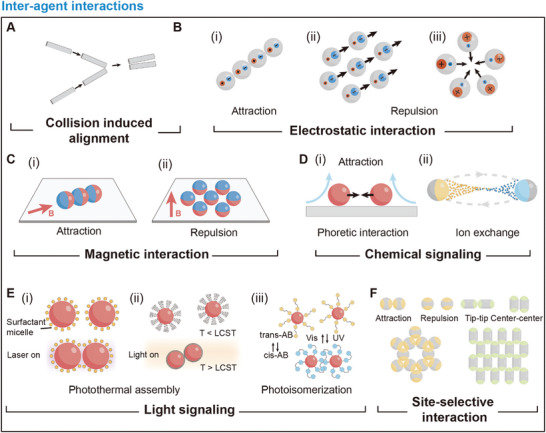
Schematic illustration of inter‐agent interactions. A) Schematics of collision‐induced alignment of self‐propelled filaments. B) Swarm generation in metal‐dielectric Janus particles through electrostatic attraction and electrostatic repulsion. C) Swarm generation in magnetic agents through i) attraction, and ii) repulsion. D) Swarm generation mediated by chemical signals, including mechanisms of i) phoretic interaction, and ii) ion exchange. E) Light‐mediated agent‐agent interaction. i) Photothermal induced assembly. ii) Photoisomerization‐induced assembly. F) Self‐assembly induced by site‐selective interactions.

Under undirected stimuli, both isotropic agents and anisotropic agents perform random locomotion. In a multiagent system, this randomness can transform into coordinated locomotion due to local velocity alignment and orientational symmetry breaking. The first model that investigates the emergence of coordinated motion in systems of active agents is the Vicsek model.^[^ ^124^ ^]^ The key concept of the Vicsek model is that in each step the constant‐speed particles adapt to take the average direction of their neighbors with random noise added. This model reproduces the kinetic phase transition from no transport to global order through velocity alignment. **Figure** **5**A provides a physical scheme of the velocity alignment of self‐propelled filaments caused by binary collision. Considering two self‐propelled rods moving longitudinally collide at an acute angle. After inelastic collision, they align parallel due to the combined effect of anisotropic repulsion and self‐propulsion. After multiple collisions in an ensemble of such active agents, local order and clustering emerge as a result of velocity alignment.^[^ ^125^ ^]^

#### 3.1.2 Electrostatic Interaction

Electrostatic forces between polarized active agents under the applied electric field can be either attractive or repulsive depending on their polarization states. By modulating electrostatic interactions and motility of active agents, various forms of swarm behaviors emerge. A typical model system for studying such interactions involves Janus particles with metal and SiO_2_ hemispheres.^[^ ^23^ ^]^ Due to the different polarization behaviors of these hemispheres, electrostatic imbalance appears across the equators of the particles, leading to particle propulsion perpendicular to the field direction. The propulsion direction is directed to the negatively charged metal side, which acts as the head of the Janus particle. The nature of the electrostatic interaction dictates the swarm generation. When two hemispheres have the same charges, an isotropic gas phase emerges, exhibiting a random configuration. When the two hemispheres have charges with different signs and similar magnitudes, polar chains appear through electrostatic attraction (Figure 5B(i)). When the negative charge on the leading metal hemisphere significantly outweighs the positive charge on the SiO_2_ hemisphere, collective motion appears due to the repulsion‐induced alignment (Figure 5B(ii)), which can be explained by the Vicsek model. Here, the reorientation of active agents is driven not by physical contact (collision) but by electrostatic repulsion. Conversely, when tail repulsion dominates, active agents tend to arrange their tails apart by forming clusters with outward‐pointing tails (Figure 5B(iii)).

#### 3.1.3 Magnetic Interaction

The swarm generation in magnetic agents mainly originates from magnetic dipole–dipole interaction. The dipolar interaction between two magnetic dipoles m_*i*_ and m_*j*_ at a separation distance r_*ij*_ = r_*i*_ − r_*j*_ can be expressed as:

(12) $$U_{m}=-\mu_{0}/4\pi \left\{\left.\left[3\left(m_{i}.r_{ij}\right)\left(m_{j}.r_{ij}\right)/r^{5}\right]-\left(m_{i}.m_{j}\right)/r^{3}\right\}\right.$$

When magnetic moments of magnetic agents are parallel to r_*ij*_, the interaction is attractive, leading to the formation of polar chains. Conversely, when perpendicular, the interaction becomes repulsive, resulting in the formation of a gas phase (Figure 5C). Paramagnetic agents, whose magnetic moments align with external magnetic fields, demonstrate various swarm behaviors when subjected to time‐varying magnetic fields. These fields can be generated by electromagnetic systems like Helmholtz coils, which enable the real‐time programming of magnetic fields in 3D space. Introducing dynamic magnetic fields like rotating magnetic fields and oscillating magnetic fields leads to the generation of magnetic swarms, such as ribbon‐like swarms and vortex‐like swarms.^[^ ^91^, ^126^, ^127^, ^128^ ^]^

Apart from direct body forces, hydrodynamic interactions also play critical roles in the generation of magnetic swarms in fluidic environments. Depending on the flow field surrounding the magnetic agents, the hydrodynamic characteristics of the agents can be categorized into different types. The combination of magnetic dipole interaction, hydrodynamic interaction, and excluded volume contributes to the emergence of various dynamic assembly and disassembly processes.^[^ ^129^ ^]^

#### 3.1.4 Chemically Mediated Inter‐Agent Interactions

##### Phoretic Interaction

In systems with reactive or catalytic active agents, chemical reactions occur on the surface of active agents in the presence of chemical fuels, leading to the generation of local phoretic flow. The inter‐agent interaction can be mediated by the local flow field. Phoretic‐induced attractions are commonly observed in isotropic catalytic colloids. In the presence of chemical fuels, the chemical reaction that happens on the catalytic colloid surface leads to the local accumulation of products, which induces an inward osmotic flow. When two agents come into proximity, the product concentration between them becomes higher, reducing the concentration gradient, and leading to attractive interaction (Figure 5D(i)). In the far‐field approximation, influenced by phoretic interaction, the drift velocity of one agent (*V*_2_) due to the activity of the other agent (*V*_1_) can be expressed as:^[^ ^130^, ^131^ ^]^

(13) $$V_{2}=-\frac{\mu_{2}}{\pi \sigma^{2}}\int dS\nabla_{\left|\right|}\left(\frac{\alpha_{1}\sigma^{2}}{4Dr}\right)=\frac{\alpha_{1}\mu_{2}\sigma^{2}}{24\pi D}\frac{r_{12}}{{\left|r_{12}\right|}^{3}}$$

where **r**_12_ = **r**_2_ − **r**_1_, *µ* is the phoretic mobility, *α* is the surface activity, *σ* is the radius of the particle, *D* is the diffusion coefficient. This mechanism also applies to systems with mixtures of active and passive agents, where passive particles can assemble around active particles into crystal structures or follow active particles driven by the phoretic flow.^[^ ^132^, ^133^ ^]^ The phoretic flow induced attraction is also observed in photoactivated colloids.^[^ ^119^, ^134^, ^135^ ^]^ Although light is used to trigger the swarm generation, the fundamental mechanism still depends on the local photochemical reaction.

##### Ion Exchange

In systems composed of chemically coupled agents, chemicals serve as signals for inter‐agent communications. For example, ion‐exchange reactions can couple self‐propelling ZnO nanorods and sulfonated PS particles, where chemical communication is established through the product exchange between two active agents (Figure 5D(ii)).^[^ ^136^ ^]^ In an ion‐exchange process, ZnO nanorods self‐propel through diffusiophoresis, while at the same time releasing Zn^2+^ and OH^−^ into the surrounding fluid. The coupling agents, sulfonated PS particles, act as ion exchangers, absorbing Zn^2+^ and releasing H^+^. This communication enhances the reactivity and motion of both ZnO nanorods and sulfonated PS particles, resulting in the generation of an active swarm of ZnO‐PS complexes. With the ion‐exchange induced inter‐agent interaction, complex swarm behaviors like phase separation emerge within this swarm.

#### 3.1.5 Light‐Induced Inter‐Agent Interactions

##### Photothermal Effect

Light can modulate inter‐agent interactions with mechanisms, such as photothermal effects and photoisomerization. The photothermal effect involves heat generation due to the photoexcitation of materials. A typical example of photothermal‐induced swarm generation is the photo‐thermophoretic flow induced assembly of inert agents. This mechanism is demonstrated in a solution of colloidal particles with ionic surfactants.^[^ ^137^ ^]^ Upon localized laser irradiation, a temperature gradient arises, leading to inward phoretic flow. The flow can trap colloidal particles at specific points and create depletion zones between adjacent particles, resulting in depletion force induced assembly (Figure 5E(i)). Another example involves agents modified with thermoresponsive polymers such as Poly(N‐Isopropylacrylamide) (PNIPAM). PNIPAM undergoes reversible hydration and dehydration accompanying hydrophilic–hydrophobic transitions. Below the critical solution temperature LCST, PNIPAM is hydrophilic. While above LCST, PNIPAM dehydrates and becomes hydrophobic, resulting in hydrophobic attraction induced aggregation or assembly (Figure 5E(ii)).^[^ ^138^ ^]^

##### Photoisomerization

Photoisomerization refers to the isomerization process induced by light. The most investigated photoisomerization process is the cis‐trans transition. Modifying nanoparticles with *trans*‐azobenzene leads to the creation of active agents with photoswitchable interactions. Upon UV irradiation, *trans*‐azobenzene isomerizes to *cis*‐azobenzene, giving rise to interparticle attraction from solvophobic interactions and dipole–dipole interactions between polar *cis*‐azobenzene.^[^ ^139^, ^140^, ^141^, ^142^, ^143^ ^]^ This attraction leads to the self‐assembly of nanoparticles, which can be reversed using UV irradiation with different wavelengths or thermal fluctuations (Figure 5E(iii)).

#### 3.1.6 Site‐Selective Inter‐Agent Interaction

Site‐selective interactions between active agents can lead to the formation of highly ordered superstructures. For example, the self‐assembly of a Kagome lattice is achieved using triblock Janus particles with two hydrophobic poles and one electrically charged middle band.^[^ ^95^ ^]^ These particles are prepared by depositing hydrophobic gold thin films on opposite poles of sulfate polystyrene microspheres. The resulting particles exhibit attractive hydrophobic interaction between their poles but electrostatic repulsion at their middle bands. The site‐selective attractive/repulsive interactions result in the formation of ordered 2D networks (Figure 5F). Directional and site‐selective bonding and assembly of the colloidal superstructure is also achieved with triblock PS‐TPM‐PS, leveraging depletion force.^[^ ^96^ ^]^ The triblock active agents are fabricated with a cluster encapsulation method, featuring a PS (polystyrene) dimer with a fused junction of TPM (polymerized 3‐(trimethoxysilyl)propyl methacrylate). Depending on the type and concentration of depletants, the interacting sites change, resulting in the formation of various superstructures.

### 3.2 Agent‐Environment Interactions

**Figure 6:**
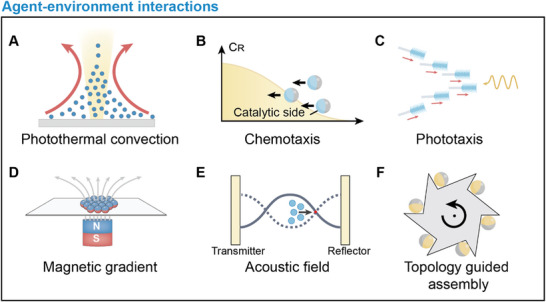
Schematic illustration of agent‐environment interactions. A) Schematics of photothermal convection induced swarm generation. B) Schematics of positive chemotaxis of catalytic active agents in a chemical concentration gradient. C) Schematics of positive phototaxis of photoactive nanotree under light illumination. C_R_ indicate the concentration of chemical fuels. D) Schematics of magnetic potential well induced swarm generation. E) Schematics of acoustic potential well induced swarm generation. F) Schematics of topology guided assembly of self‐propelling active agents.

In the presence of directed stimuli, individual active agents can perform long‐range directional migration due to directed torques and forces. Applying the same stimuli to collectives of active agents results in coherent motion. In such circumstances, the long‐range forces and torques exerted on individual agents often overwhelm inter‐agent interactions, leading to swarm behaviors like long‐range directional migration, and localized clustering (**Figure** **6**). The generation of swarms with unidirectional collective motion can be achieved through mechanisms like photothermal convection,^[^ ^144^, ^145^ ^]^ chemotaxis,^[^ ^44^, ^45^ ^]^ and phototaxis.^[^ ^38^, ^49^, ^146^ ^]^ These signals guide active agents within a swarm in a specific direction. On the other hand, clustering is often induced through specific environmental traps such as magnetic traps, acoustic traps, and topological traps.^[^ ^147^, ^148^, ^149^, ^150^, ^151^, ^152^ ^]^ These traps act as potential wells where agents gather, resulting in concentrated clusters or aggregates.

## 4 Swarm Behaviors

Swarm behaviors are broadly categorized into two primary forms: spontaneous swarm behaviors, such as self‐assembly and self‐organization, influenced by inter‐agent interactions and agent‐environment interactions, and programmed swarm behaviors, such as controlled locomotion and shape reconfiguration, achieved by introducing external programmers (typically humans or computer) to modulate interactions on‐demand. In this section, we provide a comprehensive overview of these swarm behaviors, focusing on the mechanisms underlying swarm behaviors and examining the fundamental relationship between the swarm behaviors and features of individual agents.

### 4.1 Self‐Assembly

Within this review, self‐assembly refers to the formation of highly ordered superstructures composed of active agents. These ordered assemblies can be either static or dynamic, arising from either equilibrium processes or out‐of‐equilibrium processes. However, in the context of synthetic swarm behaviors, we mainly focus on the dynamic self‐assembly of synthetic active agents in nonequilibrium systems. This section reviews various mechanisms for swarm self‐assembly.

#### 4.1.1 Directional Inter‐Agent Interaction

Directional inter‐agent interactions offer promising approaches for assembling colloidal particles into predetermined superstructures. Achieving bonding directionality typically relies on the creation of Janus particles with site‐dependent surface coating or composition. Various interactions, including electrostatic interactions, hydrophobic interactions, depletion forces, and magnetic interactions, are employed to construct colloidal superstructures. Pioneering work in this field has demonstrated the self‐assembly of a Kagome lattice using triblock Janus particles.^[^ ^95^ ^]^ These particles feature hydrophobic patterns on their poles and electrically charged middle bands, resulting in electrostatic repulsion in the middle and hydrophobic attraction at the poles. By adding salt to screen the electrostatic repulsion, the self‐assembly is initiated. The hydrophobic interaction drives the initial assembly of these agents into kinetically favored chains and irregular networks as an intermediate state, which eventually evolve into the thermodynamically favored Kagome lattice. Another example demonstrates the crystallization of biphasic colloidal particles via depletion force.^[^ ^96^ ^]^ These biphasic colloidal particles consist of polystyrene (PS) poles separated by mid‐domains of polymerized 3‐(trimethoxysilyl)propyl methacrylate (TPM). The interparticle pair‐potentials between different sites can be regulated by adjusting the depletion condition, resulting in either pole–pole attraction or center–center attraction. By fine‐tuning the interparticle interactions and particle geometries, a wealth of self‐assembled superstructures can be generated.

**Figure 7:**
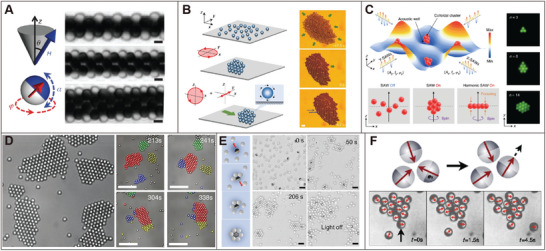
Self‐assembly in synthetic swarms. A) Magnetic field guided self‐assembly of magnetic Janus particles through synchronization. Reproduced with permission.^[^ ^93^ ^]^ Copyright 2012, Springer Nature. B) Dynamic self‐assembly of magnetic colloidal carpet guided by rotating magnetic field. Reproduced under the terms of the Creative Commons Attribution 4.0 International License (http://creativecommons.org/licenses/by/4.0).^[^ ^153^ ^]^ Copyright 2019, The authors, published by Springer Nature. C) Crystal structures formed by colloidal particles entrapped in potential wells within an acoustic field. Reproduced with permission.^[^ ^150^ ^]^ Copyright 2022, Springer Nature. D) Living crystals formed by photoactive colloidal particles under UV light via phoretic interaction. Reproduced with permission.^[^ ^134^ ^]^ Copyright 2013, American Association for the Advancement of Science. E) Living crystal formed by photoactive colloidal particles and same‐sized inert particles. Reproduced with permission.^[^ ^132^ ^]^ Copyright 2017, Wiley‐VCH. F) Motility‐induced self‐assembly of self‐propelled colloidal particles. Reproduced with permission.^[^ ^154^ ^]^ Copyright 2013, American Physical Society.

While the previous examples focus on building blocks undergoing random Brownian motion, we now look into the dynamic self‐assembly of active agents exhibiting periodic motions. A key aspect here is synchronization, where individual agents, each performing periodic motion with adjustable phase and frequency, couple their motions. To achieve synchronization, phase freedom of motion is required for active agents to adjust their phases through interparticle interactions. One notable example involves the self‐assembly of magnetic Janus particles into microtube structures.^[^ ^93^ ^]^ This process involves the implementation of a programmed dynamic magnetic field to magnetic particles with discoid magnetic symmetry, promoting magnetic particles to perform the gyroscope's nutation. When two agents approach each other closely (within ≈200 nm), the magnetic fields generated by these agents exert appreciable torques on their neighboring agents, gradually leading to synchronous movement and large‐scale microtube formation (**Figure** **7**A).

#### 4.1.2 Magnetic Field Induced Dynamic Self‐Assembly

Apart from the synchronization induced self‐assembly, time‐varying magnetic fields have also been used to guide the dynamic self‐assembly of isotropic paramagnetic colloidal particles. Applying rotating magnetic fields in the plane of the substrate with sufficiently high frequencies results in time‐averaged isotropic and attractive interactions between magnetic particles, leading to the formation of crystal structure from randomly dispersed magnetic particles (Figure 7B).^[^ ^153^ ^]^ Under the rotating magnetic field, the crystal performs a spinning motion around its center due to the net magnetic torque.

#### 4.1.3 Surface Functionalization Mediated Self‐Assembly

Stimuli‐responsive surface functionalization enables the on‐demand formation of chemical bonds between surface linkers, thereby inducing the self‐assembly of active agents. Utilizing DNA for functionalizing active agents is promising in programming the self‐assembly of active agents. DNA‐functionalized active agents can attract each other through DNA hybridization.^[^ ^155^ ^]^ For example, gold nanoparticles functionalized with DNA oligonucleotides reversibly aggregate and disassemble in response to temperature changes, driven by temperature‐dependent formation and melting of the DNA bridge.^[^ ^156^ ^]^ Based on the programmed interaction, temperature‐encoded self‐assembly of active agents is achieved using emulsion droplets functionalized with DNA strands.^[^ ^157^ ^]^

Photoresponsive molecules like azobenzene which reversibly isomerize between different states under light of different wavelengths, are also used for mediating interparticle interactions. The transition between nonpolar and polar states of azobenzene induces attraction and self‐assembly of azobenzene‐modified nanoparticles in a nonpolar solvent.^[^ ^141^ ^]^ Moreover, by finely tuning interparticle interactions, it is also possible to achieve self‐assembled spherical aggregates in addition to 2D crystal‐like assemblies.^[^ ^140^ ^]^ Another light‐induced self‐assembly approach involves functionalizing nanoparticles with coumarins, which undergo cycloaddition reactions when exposed to UV light (365 nm). Leveraging this photodimerization reaction, the reversible assembly of coumarins coated gold nanoparticles has been achieved.^[^ ^158^, ^159^ ^]^

#### 4.1.4 Potential Well Induced Self‐Assembly

Potential wells are regions in space where potential energy is lower than surrounding areas. These wells are typically created using external stimuli that asymmetrically interact with active agents. For instance, when subjected to a magnetic field gradient, magnetic agents experience a force directed toward the field gradient. In this scenario, regions with the maximum field strength are considered magnetic potential wells. These wells have been used to create assemblies of magnetic agents with well‐defined contours on liquid–air interface with the assistance of surface tension.^[^ ^160^ ^]^ By tuning the magnetic field, both the shape reconfiguration and magnet‐driven locomotion of these assemblies can be achieved. Another method for achieving desired potential wells involves utilizing surface acoustic waves (SAWs). Potential wells can be generated by applying surface acoustic waves with finely tuned frequency and amplitude in an orthogonal arrangement.^[^ ^150^ ^]^ Particles can be trapped in acoustic wells, where they experience minimized acoustic radiation forces. This leads to the aggregation of colloidal particles within these wells and the formation of diverse crystal structures, driven by secondary acoustic radiation forces induced by scattering of acoustic waves (Figure 7C).^[^ ^161^ ^]^ Apart from magnetic potential wells and acoustic potential wells, self‐assembly is also observed in potential wells induced by optical fields.^[^ ^162^, ^163^ ^]^

#### 4.1.5 Phoretic Interaction Induced Self‐Assembly

As previously mentioned, catalytic active and ion‐exchange agents attract their peer or inert agents through phoretic interactions. Phoretic attraction induced self‐assembly has been observed in various systems, including platinum‐coated gold nanoparticles,^[^ ^164^, ^165^ ^]^ hematite cube embedded colloidal spheres,^[^ ^134^ ^]^ and iridium‐based Janus particles.^[^ ^12^ ^]^ In these systems, platinum, hematite, and iridium act as catalysts, decompose chemical fuels such as H_2_O_2_ and hydrazine to provide propulsion. Figure 7D shows the self‐assembly of hematite cube embedded colloidal particles.^[^ ^134^ ^]^ Under blue light, these particles form crystallites driven by phoretic attraction. These structures actively translate, rotate, collide, join, and split, demonstrating dynamic behaviors due to the out‐of‐equilibrium nature of the colloidal particles. The similar living crystals have also been observed in the colloidal system consisting of photoactive Janus particles (TiO_2_/SiO_2_) and size‐matched inert silica particles.^[^ ^132^ ^]^ Under UV light, active particles won't aggregate, but inert particles bond to active particles and initiate the dynamic self‐assembly, resulting in living crystals of active‐passive mixtures (Figure 7E). The self‐assembly of ion‐exchange particles has also been extensively investigated, demonstrating phoretic flow induced long‐range interactions.^[^ ^166^, ^167^ ^]^ One noteworthy feature of phoretic flow induced self‐assembly is that the formed crystal structures are usually dynamic. These structures can merge, break apart, or dissolve due to the out‐of‐equilibrium collisions of the self‐propelled particles.

#### 4.1.6 Motility Induced Self‐Assembly

In contrast to attraction‐induced assembly, self‐assembly can occur in active agents without inherent attractions through motility‐related mechanisms, such as self‐trapping.^[^ ^154^, ^168^ ^]^ As illustrated in Figure 7F, when self‐propelled particles collide head‐on, they block each other, forming a temporary cluster. The cluster disassembles on a time scale determined by rotational diffusion time, which is ≈1/*D_r_*, with *D_r_* representing the rotational diffusion coefficient. During this period, other particles may collide with the cluster and trapped within it. As a result, the stability of these clusters is determined by the balance between incoming and outgoing particles, influenced by factors like velocity, density, and rotational diffusion time. This mechanism leads to the formation of dynamic living crystals, where the size and stability of clusters are dependent on the interplay of these factors.^[^ ^154^ ^]^

### 4.2 Self‐Organization

Self‐organization arises from the coordination and velocity alignment of individual agents. In this review, we categorize self‐organization behaviors into three primary forms: clustering, vortex formation, and swarming. This section focuses on the mechanisms underlying the self‐organization of active agents.

#### 4.2.1 Clustering

**Figure 8:**
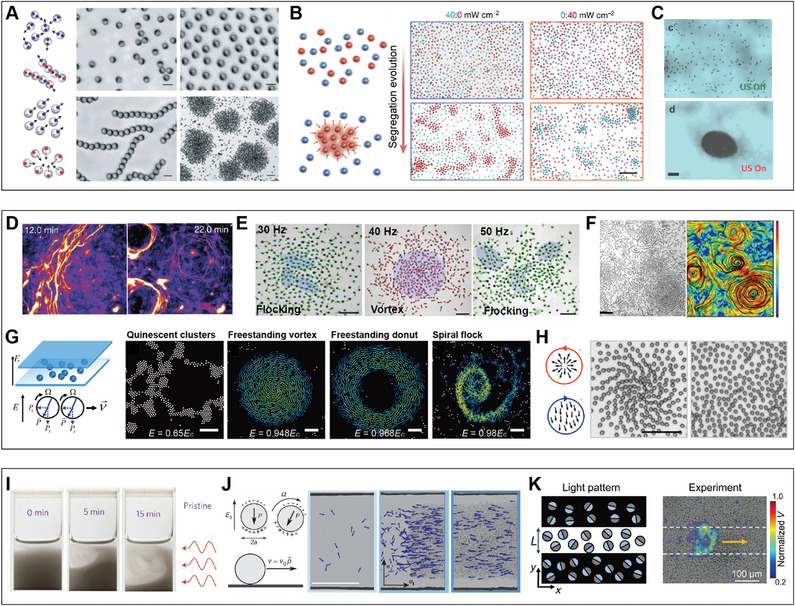
Self‐organization in synthetic swarms. A) Swarm behaviors observed in collectives of metal‐dielectric Janus particles under AC electric field. Reproduced with permission.^[^ ^23^ ^]^ Copyright 2016, Springer Nature. B) Wavelength‐selective clustering of dye‐sensitized TiO_2_ colloidal particles under illumination. Reproduced under the terms of the Creative Commons Attribution 4.0 International License (http://creativecommons.org/licenses/by/4.0).^[^ ^119^ ^]^ Copyright 2023, The authors, published by Springer Nature. C) Ultrasound‐induced clustering of chemically powered Au‐Pt Janus nanorods. Reproduced with permission.^[^ ^169^ ^]^ Copyright 2015, American Chemical Society. D) Vortex formation of microtubules on dynein‐grafted substrate in the presence of ATP. Reproduced with permission.^[^ ^170^ ^]^ Copyright 2012, Springer Nature. E) Vortex formation of magnetic rollers on a slightly concave substrate under uniaxial alternating magnetic fields. Reproduced under the terms of the Creative Commons Attribution‐NonCommercial License.^[^ ^7^ ^]^ Copyright 2017, The authors, published by American Association for the Advancement of Science. F) Multivortex formation of the magnetic roller on the flat substrate under a uniaxial alternating magnetic field. Reproduced under the terms of the PNAS license.^[^ ^171^ ^]^ Copyright 2020, National Academy of Sciences. G) Vortex formation of Quincke rollers in a DC electric field. Reproduced under the terms of the PNAS license.^[^ ^172^ ^]^ Copyright 2021, National Academy of Sciences. H) Vortex formation of anisotropic Quincke rollers in a DC electric field. Reproduced under the terms of the Creative Commons Attribution 4.0 International License (http://creativecommons.org/licenses/by/4.0).^[^ ^173^ ^]^ Copyright 2020, The authors, published by Springer Nature. I) Phototaxis of phototactic microswimmers under light illumination. Reproduced with permission.^[^ ^38^ ^]^ Copyright 2016, Springer Nature. J) Directed motion of Quincke rollers through collision‐induced alignment. Reproduced with permission.^[^ ^4^ ^]^ Copyright 2013, Springer Nature. K) Propagating wave in chemically active silver colloids. Reproduced under the terms of the Creative Commons Attribution‐NonCommercial License.^[^ ^174^ ^]^ Copyright 2022, The authors, published by American Association for the Advancement of Science.

Clustering refers to the phenomenon where aggregates emerge from an initially randomly distributed population of active agents. This phenomenon is common in nature, such as the formation of bird flocks and fish schools.^[^ ^175^, ^176^, ^177^ ^]^ These behaviors could be resulted from the response of individual agents to their peers, via signals such as chemicals, visual perception, and acoustic signals, or guided by environmental signals.^[^ ^178^, ^179^, ^180^, ^181^ ^]^ The clustering can also be realized in synthetic swarms through applying inter‐agent interactions and environmental stimuli. Inter‐agent interaction induced clustering has been observed in metal‐dielectric Janus particles.^[^ ^23^ ^]^ Metal‐dielectric Janus particles exhibit an electrostatic imbalance when subjected to alternating electric fields, causing them to perform electrophoresis toward the metal side. When the dipolar interaction is adjusted within a specific range, jammed clusters of high local density form due to the collision and reorientation of particles. Further adjustments of the frequency and amplitude of the alternating electric field lead to other forms of self‐organization, such as flocking and chain formation, within this system (**Figure** **8**A).

Photochemical reaction induced phoretic flow has been harnessed to mediate mutual interactions between photoactive agents.^[^ ^132^, ^134^, ^182^ ^]^ The phoretic flow fields typically establish both long‐range attraction and short‐range repulsion between active agents. By tuning the pair potentials and selectively activating photoactive agents with specific light adsorptions, controlled clustering of active agents has been achieved (Figure 8B).^[^ ^119^ ^]^ Similar phoretic interaction has also been found to facilitate the clustering of catalytic active agents.^[^ ^183^ ^]^ In situations where local potential wells exist, active agents are driven toward these potential wells to minimize their potential energy, forming clusters. Examples of such behavior can be found in systems with magnetic field gradients or acoustic traps.^[^ ^152^, ^169^ ^]^ For instance, using an acoustic trap formed in standing acoustic waves, reversible clustering of chemically powered nanomotors can be achieved (Figure 8C).

#### 4.2.2 Vortex Formation

The formation of vortex in nature, i.e., active turbulence, can be found in a wide range of biological systems, such as bacteria swarms, sperm swarms, tissue cell monolayers, and microtubule‐kinesin collectives.^[^ ^184^, ^185^, ^186^, ^187^, ^188^, ^189^, ^190^ ^]^ The emergence of turbulent motion is mainly driven by the self‐propulsion and mutual interaction of active agents, and has important effects on nutrient mixing and molecular transport in microbiological systems.^[^ ^188^, ^191^, ^192^ ^]^

The self‐organization of active agents into the vortex is observed in various systems, including microtubules, Quincke rollers, and magnetic rollers.^[^ ^36^, ^170^, ^193^ ^]^ The formation of these vortices primarily results from velocity alignment. Microtubules, for instance, can be propelled by surface‐bound dynein in the presence of ATP, exhibiting reptation‐like motion. As a result of self‐propulsion and collision‐induced nematic alignment, microtubules in high densities self‐organize into vortices (Figure 8D).^[^ ^170^ ^]^ Such vortices formation observed in microtubules greatly resembles the active turbulence exhibited by bacteria colonies.^[^ ^188^ ^]^

Energized by uniaxial alternating magnetic fields, ferromagnetic rollers form vortices on a slightly concave substrate.^[^ ^7^ ^]^ The emergence of the vortex is an outcome of collision and magnetic interaction induced synchronization. By adjusting the frequency of the alternating magnetic field, the motility of rollers can be modulated, thereby generating other swarm behaviors like flocking (Figure 8E). Without the guidance of a concave substrate, the emergence of multivortex state in ferromagnetic nickel spheres in an open environment is also demonstrated, which is induced by dynamic local roller densification (Figure 8F).^[^ ^171^ ^]^

Vortex formation is also reported in systems of Quincke rollers energized by DC electric fields. The coherent motion of Quincke rollers emerges under a DC electric field with an amplitude larger than a threshold value, *Ec*.^[^ ^4^ ^]^ However, a study reveals different phenomena occurring below this threshold, in the subcritical regime, where *E* < *Ec*.^[^ ^172^ ^]^ The self‐organization is resulted from a combination of local subcriticality, activity, and hydrodynamics. By modulating the activity of Quincke rollers through changing the amplitude of the electric field, a range of swarm behaviors can be achieved, such as excitation wave, vortex, and spiral flock (Figure 8G). Additionally, leveraging the self‐propelling nature of Quincke rollers, shape‐anisotropic rollers with inherent curved trajectories have been developed.^[^ ^173^ ^]^ The complexity introduced by the chiral structure significantly contributes to the vortex formation (Figure 8H). Generally, the vortex generation in self‐organizing systems demands greater complexity than behaviors like flocking and clustering. The complexity often arises from factors, such as geometry confinement, hydrodynamic interaction, and shape anisotropy. Integrating these complex elements reshapes the communication network between active agents, leading to the emergence of complex and dynamic swarm behaviors.

#### 4.2.3 Swarming

Swarming refers to the directed collective migration of active agents. In living organisms, swarming is commonly realized through tactic response, which originates from the response of individual organisms to environmental stimuli. According to the types of stimuli, taxis behaviors can include chemotaxis,^[^ ^194^, ^195^, ^196^, ^197^, ^198^ ^]^ phototaxis,^[^ ^199^ ^]^ rheotaxis,^[^ ^200^, ^201^, ^202^, ^203^ ^]^ and magnetotaxis.^[^ ^199^ ^]^ In synthetic swarms, swarming can be induced by both directed and undirected stimuli. Directed stimuli, such as rotating magnetic fields or asymmetrical light illumination, can effectively induce swarming by guiding the coherent motion of active agents in specific directions.^[^ ^204^ ^]^ For example, under asymmetrical light illumination, chemically modified self‐propelled Janus nanotrees can perform either positive or negative phototaxis, schooling toward or away from the light source (Figure 8I).^[^ ^38^ ^]^ Swarming in response to undirected stimuli is particularly fascinating for understanding complex swarm behaviors. An example of such behavior involves the swarming of Quincke rollers in a confined channel. Governed by short‐range hydrodynamic interactions, self‐propelling Quincke rollers align their velocities. Complex collisions and coalescence lead to the emergence of a polar band propagating at a constant velocity (Figure 8J).^[^ ^4^ ^]^

Swarming is also observed in photochemically active silver‐containing colloids.^[^ ^174^ ^]^ When subjected to UV illumination and chemical fuels, Ag Janus particles exhibit oscillatory propulsion, alternating between active and resting stages. In dense populations of Ag Janus particles, swarming waves emerge, driven by a traveling chemical wave, primarily consisting of OH^−^ ions from reaction‐diffusion processes. As the chemical wave propagates, the local pH at the wavefront increases, which enhances catalytic reactions and creates a positive feedback loop. The feedback mechanism results in rigorous chemical reactions at the wavefront, triggering the fast motion of Ag Janus particles, and further propagating the wave through reaction‐diffusion mechanisms (Figure 8K).

### 4.3 Toward Designing Swarm Behaviors

**Figure 9:**
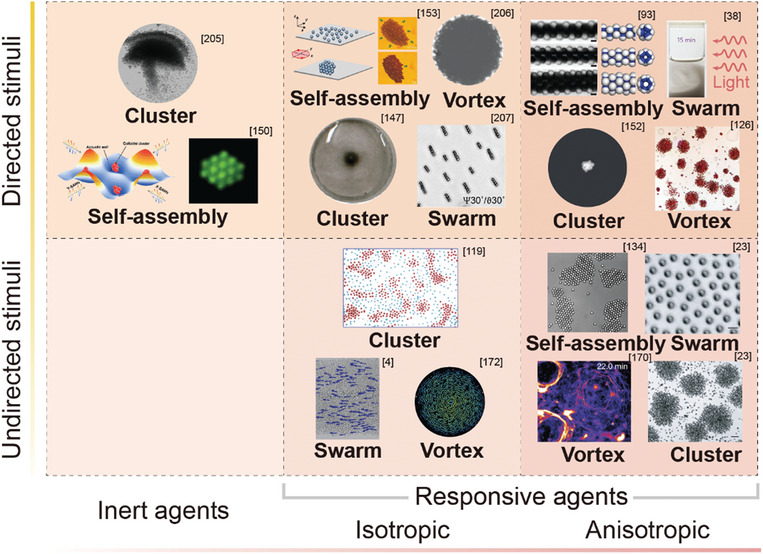
Summary of emergent swarm behaviors in synthetic swarms of active agents with distinct properties under different external stimuli. Cluster formation induced by ultrasound. Reproduced with permission.^[^ ^205^ ^]^ Copyright 2020, Wiley‐VCH. Self‐assembly induced by acoustic waves. Reproduced with permission.^[^ ^150^ ^]^ Copyright 2022, Springer Nature. Self‐assembly induced by the rotating magnetic field. Reproduced under the terms of the Creative Commons Attribution 4.0 International License (http://creativecommons.org/licenses/by/4.0).^[^ ^153^ ^]^ Copyright 2019, The authors, published by Springer Nature. Vortex induced by the rotating magnetic field. Reproduced with permission.^[^ ^206^ ^]^ Copyright 2022, IEEE. Cluster formation induced by the magnetic field gradient. Reproduced with permission.^[^ ^147^ ^]^ Copyright 2019, American Association for the Advancement of Science. Swarm of magnetic microparticles. Reproduced under the terms of the Creative Commons Attribution License.^[^ ^207^ ^]^ Copyright 2019, The authors, published by Wiley‐VCH. Self‐assembly of magnetic Janus particles. Reproduced with permission.^[^ ^93^ ^]^ Copyright 2012, Springer Nature. Swarm of phototactic microswimmers under illumination. Reproduced with permission.^[^ ^38^ ^]^ Copyright 2016, Springer Nature. Cluster formation of active Janus particles. Reproduced under the terms of the Creative Commons Attribution 4.0 International License (http://creativecommons.org/licenses/by/4.0).^[^ ^152^ ^]^ Copyright 2016, The authors, published by Springer Nature. Vortex of peanut‐shaped magnetic agents. Reproduced with permission.^[^ ^126^ ^]^ Copyright 2019, American Association for the Advancement of Science. Clustering of dye‐sensitized TiO_2_ colloidal particles under illumination. Reproduced under the terms of the Creative Commons Attribution 4.0 International License (http://creativecommons.org/licenses/by/4.0).^[^ ^119^ ^]^ Copyright 2023, The authors, published by Springer Nature. Swarmming of Quincke rollers through collision‐induced alignment. Reproduced with permission.^[^ ^4^ ^]^ Copyright 2013, Springer Nature. Vortex formation of Quincke rollers in a DC electric field. Reproduced under the terms of the PNAS license.^[^ ^172^ ^]^ Copyright 2021, National Academy of Sciences. Living crystals formed by photoactive colloidal particles under UV light via phoretic interaction. Reproduced with permission.^[^ ^134^ ^]^ Copyright 2013, American Association for the Advancement of Science. Vortex formation of microtubules on dynein‐grafted substrate in the presence of ATP. Reproduced with permission.^[^ ^170^ ^]^ Copyright 2012, Springer Nature. Swarming and clustering of Janus particles induced by electrostatic imbalance. Reproduced with permission.^[^ ^23^ ^]^ Copyright 2016, Springer Nature.

In this review, spontaneous swarm behaviors, including self‐assembly and self‐organization, are seen as behaviors that spontaneously emerge in a group of active agents given static or periodic stimuli, without human intervention. The emergence of these behaviors is dependent on the features of active agents and the nature of external stimuli. External stimuli can be classified into two types based on their influence on active agents: undirected stimuli, which energize active agents, resulting in random motion, and directed stimuli, which apply preset force and torque on active agents, leading to directional propulsion. Agents are categorized into inert agents and responsive agents. Inert agents do not change their properties (physical or chemical) when subjected to external stimuli. Responsive agents, on the other hand, change their properties in response to specific external stimuli. For example, paramagnetic particles polarize in external magnetic fields, and catalytic agents prompt chemical reactions in the presence of chemical fuels. We correlate reported swarm behaviors with the types of active agents and external stimuli, as illustrated in **Figure** **9** and **Table** **1**. We observe that most swarm behaviors emerge predominately in responsive agents. Inert agents self‐assemble or aggregate into ordered structures only under certain conditions. For example, inert agents without mutual interactions form clusters or crystal structures in the presence of acoustic fields. However, as agents become responsive, exhibiting increased complexity, versatile other behaviors such as vortex formation and swarming emerge. Furthermore, the versatility of swarm behaviors can be further enriched by adopting anisotropic agents with greater complexity. We also conclude that swarm behaviors under directed stimuli are more predictable and controllable compared to those under undirected stimuli. For example, the direction of swarming and the chirality of the vortex are mostly random under undirected stimuli but become controllable under directed stimuli. This controllability opens new opportunities for constructing microrobotic swarms with enhanced maneuverability and functionality.

**Table 1:** Summary of swarm behaviors.

Stimuli		Agents		Swarm behaviors	Refs.
Undirected stimuli	DC electric field	Isotropic/responsive agents	PMMA microsphere	Swarm	[4]
	DC electric field		PS microsphere	Vortex	[172]
	Light		Dye‐sensitized SiO_2_	Cluster	[119]
	Light	Anisotropic/responsive agents	Hematite cube/TPM	Self‐assembly	[134]
	Light		Ir/SiO_2_ Janus particle	Cluster	[12]
	AC electric field		Ti/SiO_2_ Janus particle	Swarm/Cluster/Vortex	[23]
	ATP		Microtubule	Vortex	[170]
Directed stimuli	Acoustic field	Inert agents	Colloidal particle	Self‐assembly	[150]
			EGaIn nanorod	Cluster	[205]
	Rotating magnetic field	Isotropic/responsive agents	Paramagnetic colloidal particle	Self‐assembly	[153]
	Magnetic field gradient		Iron oxide NP	Cluster	[147]
	Rotating magnetic field		Iron oxide NP	Vortex	[127, 206]
	Rotating magnetic field		Superparamagnetic PS microparticle	Swarm	[207]
	Processing magnetic field	Anisotropic/responsive agents	Ni/SiO_2_ Janus particle	Self‐assembly	[93]
	Acoustic field		Pt/PS Janus particle	Cluster	[152]
	Rotating magnetic field		Peanut‐shaped magnetic particle	Vortex / Swarm	[126]
	Light		Janus nanotree	Swarm	[38]

### 4.4 Toward Simulating Swarm Behaviors

**Figure 10:**
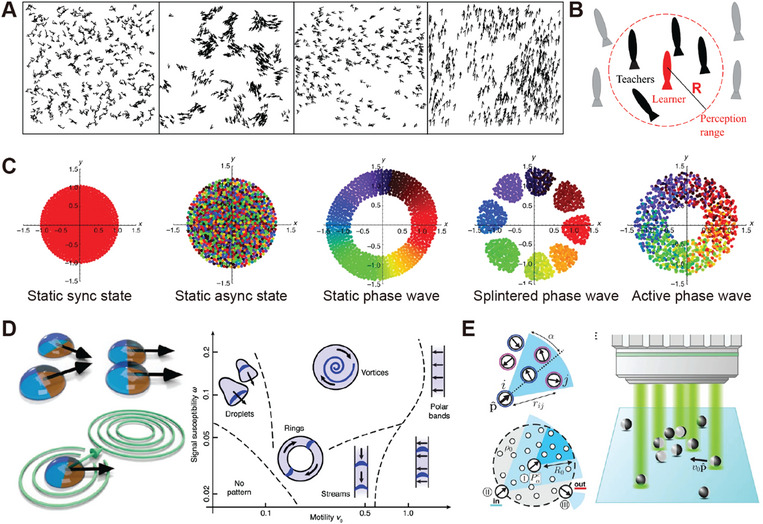
Computational studies on swarm behaviors. A) The phase transition of self‐propelling active agents simulated by the Vicsek model. Reproduced with permission.^[^ ^124^ ^]^ Copyright 1995, American Physical Society. B) Multiagent reinforcement learning on flocking behavior. Reproduced with permission.^[^ ^208^ ^]^ Copyright 2020, American Physical Society. C) Simulated swarm behaviors of oscillating active agents. Reproduced under the terms of the Creative Commons Attribution 4.0 International License (http://creativecommons.org/licenses/by/4.0).^[^ ^209^ ^]^ Copyright 2017, The authors, published by Springer Nature. D) Simulated phase diagram of the synthetic swarm that self‐organizes through long‐range inter‐agent interactions. Reproduced under the terms of the Creative Commons Attribution 4.0 International License (http://creativecommons.org/licenses/by/4.0).^[^ ^210^ ^]^ Copyright 2022, The authors, published by Springer Nature. E) Active agents with perception‐dependent motility. Reproduced with permission.^[^ ^181^ ^]^ Copyright 2019, American Association for the Advancement of Science.

In addition to experimental observation of swarm behaviors, computational studies are also extensively conducted to provide a deeper understanding of swarm behaviors. These computational studies mostly utilize agent‐based models, which offer a generative approach to investigating the evolution of swarm patterns.^[^ ^211^, ^212^ ^]^ Rather than the forces and mechanics at the individual agent level, this approach focuses more on the order and fluctuations of an entire active agent system. The Vicsek model is a seminal agent‐based model used for investigating phase transitions in swarms, which describes systems composed of self‐propelling agents with constant speeds.^[^ ^124^ ^]^ These active agents adjust their moving direction to align with that of their neighbors at each time step. The Vicsek model reveals the phase transition from the disordered phase to the ordered phase when the agent density is high or the noise level is low (**Figure** **10**A). Taking factors such as symmetry of active agents, inter‐agent interactions, and hydrodynamic interactions into consideration, the Vicsek model can be further extended.^[^ ^213^, ^214^, ^215^ ^]^ The phase transition predicted by the Vicsek model can also be investigated by multiagent reinforcement learning, which can be used to train agents to follow leader agents or form a swarm in the absence of leader agents (Figure 10B).^[^ ^208^ ^]^ The emergent navigation strategy in these scenarios aligns well with the velocity alignment rule of the Vicsek model.

Computational models capable of simulating complex swarm behaviors have been developed. By coupling the phase and spatial dynamics of oscillating active agents, five collective states can be predicted based on a generalized model (Figure 10C).^[^ ^209^ ^]^ By adding more realistic features to oscillating active agents, such as local coupling, nonidentical natural frequencies, and chirality, new swarm behaviors have been predicted, for example, lattices of vortices, beating clusters, and phase waves.^[^ ^216^ ^]^

Besides local interactions between active agents, such as agent–agent collision and velocity alignment, long‐distance interactions like chemical communications also play an important role in regulating swarm behaviors. To investigate the agent–agent communication in self‐organization process, simulated agents that can produce and propagate signals mimicking the chemical signaling process have been proposed (Figure 10D).^[^ ^210^ ^]^ It revealed that the signal transduction network contributes to the emergence of various swarm behaviors, and systematically investigated the phase diagram governed by signal susceptibility and the motility of active agents. This computational study provides insights into the swarm behavior of active agents possessing on‐board information processing capabilities.

The interaction rule of synthetic active agents can be pre‐defined, and the resulting swarm can emulate the natural swarm behaviors and verify the rules that dominate them. Swarms in nature, such as bird flocks, are formed by the motion adjustment of individuals in response to the visual perception of their peers. Such behavior can be replicated with synthetic swarms by assigning each synthetic active agent with a simulated visual perception range and individually laser‐activated motility (Figure 10E).^[^ ^217^ ^]^ When the number of peer agents in the perception range of an active agent is above a predefined threshold, the active agents are programmed to be activated with an external controller. The perception‐dependent motion adjustment leads to the aggregation of synthetic active agents. By applying predefined interaction rules to individually controllable synthetic active agents, other swarm behaviors, for example, the spontaneous vortex formation, are also validated.^[^ ^218^ ^]^ In summary, with computational studies, physical rules behind complex swarm behaviors have been investigated and validated theoretically. These studies provide a profound understanding of swarm behaviors observed in natural or synthetic swarms. Based on these findings, swarm behaviors could be predicted or designed in synthetic swarms.

## 5 Swarm Autonomy and Machine Intelligence

The investigation of the underlying mechanisms behind swarm behaviors of synthetic swarms unveils profound insights into understanding the swarm behavior observed in nature, ranging from the formation of bird flocks to the emergence of bacterial colonies. Beyond investigating self‐organization within ensembles of active agents that focus on interaction at the individual level, researchers also investigate into the behaviors of these swarms as entities. The shape reconfiguration and locomotion capabilities of these swarms make them promising entities for constructing microrobots.^[^ ^219^ ^]^ The controlled locomotion of microrobotic swarms has been extensively investigated in the past decade and substantial progress has been made. The abilities of microrobotic swarms to follow external instructions or environmental stimuli further provide possibilities to infuse machine intelligence into synthetic swarms to achieve swarm autonomy. In this section, we provide a summary of the controlled locomotion of microrobotic swarms and discuss the realization of swarm autonomy through integrating machine intelligence.

### 5.1 Controlled Locomotion

While extensive research has been conducted on the swarm behaviors of synthetic swarms, transforming these swarms into functional microrobotic systems faces several challenges. These challenges include limitations in mobility, integrity, and actuation system compatibility. The reported systems exhibiting controlled locomotion predominantly rely on actuation methods like magnetic field, acoustic field, and light. In this session, we provide an overview of existing microrobotic swarms and their capabilities.

**Figure 11:**
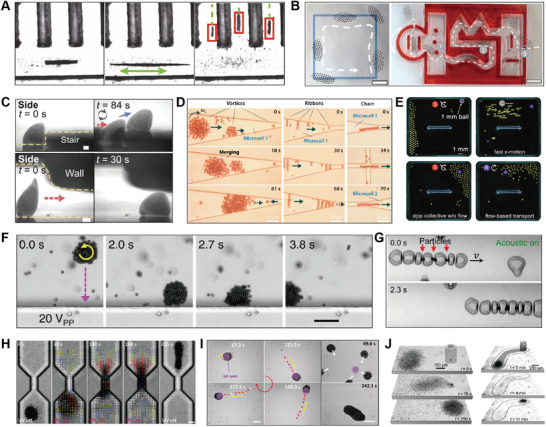
Controlled locomotion of microrobotic swarms. A) Elongation and splitting of ribbon‐like swarms for moving toward different destinations. Reproduced under the terms of the Creative Commons Attribution 4.0 International License (http://creativecommons.org/licenses/by/4.0).^[^ ^91^ ^]^ Copyright 2018, The authors, published by Springer Nature. B) Locomotion and reconfiguration of a microrobotic swarm formed by magnetic particles for passing through a complex environment. Reproduced under the terms of the Creative Commons Attribution 4.0 License.^[^ ^160^ ^]^ Copyright 2020, The authors, published by SAGE. C) A vertical microrobotic swarm is actuated to climb up a stair and reconfigures to adapt to a confined environment. Reproduced under the terms of the Creative Commons Attribution 4.0 License.^[^ ^221^ ^]^ Copyright 2022, The authors, published by American Association for the Advancement of Science. D) Locomotion of microrobotic swarms with different configurations in narrow channels. Reproduced with permission.^[^ ^126^ ^]^ Copyright 2019, American Association for the Advancement of Science. E) Object manipulation by a swarm of magnetic disks at the water‐air interface. Reproduced under the terms of the Creative Commons Attribution 4.0 International License (http://creativecommons.org/licenses/by/4.0).^[^ ^104^ ^]^ Copyright 2022, The authors, published by Springer Nature. F) A magnetic swarm approaches and rolls along the boundary of a vessel under a rotating magnetic field and an acoustic field. Reproduced under the terms of the Creative Commons Attribution 4.0 International License (http://creativecommons.org/licenses/by/4.0).^[^ ^222^ ^]^ Copyright 2017, The authors, published by Springer Nature. G) Particle transportation achieved by a train‐like swarm consisting of bubbles actuated by an acoustic field. Reproduced under the terms of the Creative Commons Attribution 4.0 International License (http://creativecommons.org/licenses/by/4.0).^[^ ^223^ ^]^ Copyright 2023, The authors, published by Springer Nature. H) A phototactic swarm passes through a narrow channel actuated by light. Reproduced under the terms of the Creative Commons Attribution 4.0 International License.^[^ ^224^ ^]^ Copyright 2019, The authors, published by Cell Press. I) Navigation and merging of TiO_2_ swarms using UV light. Reproduced with permission.^[^ ^225^ ^]^ Copyright 2023, American Chemical Society. J) Transportation of a colloidal swarm by optical and electric fields. Reproduced with permission.^[^ ^226^ ^]^ Copyright 2014, Wiley‐VCH.

Magnetic fields, generated by electromagnetic coils or permanent magnets, offer promising actuation methods for magnetic swarms.^[^ ^220^ ^]^ The use of three‐axis Helmholtz coils, for example, enables the creation of time‐varying uniform magnetic fields in 3D space. Actuated by these fields, magnetic swarms with various configurations are created, such as ribbon‐like swarms and vortex‐like swarms.^[^ ^91^, ^127^ ^]^ By adjusting actuation parameters, these magnetic swarms can dynamically reconfigure and execute directed locomotion. **Figure** **11**A illustrates the adaptive locomotion of a ribbon‐like magnetic swarm composed of iron oxide nanoparticles, demonstrating its ability to elongate and split into three distinct parts for exploring different channels.^[^ ^91^ ^]^ Utilizing permanent magnets, magnetic disks can form specific patterns and navigate along predetermined trajectories at the liquid–air interface, driven by magnetic field gradient.^[^ ^160^ ^]^ The magnetic swarm exhibits remarkable adaptability, capable of reconfiguring to navigate cluttered environments with obstacles (Figure 11B).

While most magnetic swarms are confined to a 2D plane, either settling on the substrate or being trapped by the liquid–air interface, recent advances have led to the development of vertical magnetic swarms. These swarms are generated by tailoring inter‐particle interactions of magnetic particles using a unique dual‐axis oscillating magnetic field.^[^ ^221^ ^]^ The vertical swarm exhibits remarkable capabilities, such as collaboratively ascending steep staircases and dynamically reconfiguring to navigate through a channel with varying heights (Figure 11C). Beyond spherical agents, peanut‐shaped magnetic particles have also emerged as promising elements for reconfigurable microrobotic swarms.^[^ ^126^ ^]^ The reconfiguration capability greatly enhances the adaptability of these swarms to changing environments. For example, swarms with vortex and ribbon‐like configurations cannot effectively pass through narrow channels, due to the geometry confinement. While chain‐like swarms can smoothly pass through narrow channels (Figure 11D). The shape‐reconfigurable magnetic swarms can also mimic natural swarm behaviors like cargo transportation, as seen in ant colonies. Floating magnetic disks, for example, can coordinate to surround and strategically maneuver objects, demonstrating potential in coordinated cargo handling (Figure 11E).^[^ ^104^ ^]^

Combining magnetic and acoustic fields has led to an innovative propulsion method. With this approach, clusters of magnetic particles can form under a rotating magnetic field, and an acoustic field can be used to transport the rolling cluster to the vessel boundary for more efficient propulsion (Figure 11F).^[^ ^222^ ^]^ Moreover, by manipulating the acoustic nodes within a vessel, effective upstream propulsion of magnetic swarms is achieved.^[^ ^227^ ^]^ This propulsion mechanism offers a promising strategy for applications like targeted drug delivery via the vascular system. The acoustic field, when utilized independently, also exhibits remarkable potential for controlling microrobotic swarms. Acoustic fields can energize a wide array of agents with distinct features, including microbubbles,^[^ ^149^ ^]^ cells,^[^ ^52^, ^54^, ^150^ ^]^ Janus particles,^[^ ^152^ ^]^ and nanorods.^[^ ^169^ ^]^ An example is the acoustic field controlled microbubble swarm (Figure 11G). The train‐like microbubble swarm is generated within a viscous fluid confined between a glass slide and a capillary tube, driven by the amplified acoustic radiation pressure within the narrow slit. This arrangement enables efficient cargo transportation within confined spaces.^[^ ^223^ ^]^

In contrast to magnetic fields and acoustic fields, which provide globally directed stimuli to microrobotic swarms, light offers distinct control mechanisms, either through phototaxis or photothermal convection flow. Photocatalytic microrobotic swarms, for example, can be navigated by phototaxis.^[^ ^224^ ^]^ Hydroxyl‐modified TiO_2_ micromotors spontaneously aggregate in aqueous media due to electrolyte diffusiophoretic attractions. Upon being illuminated with incident light, the swarm undergoes expansion and moves away from the incident light. Under alternating light illumination, the swarm expands and contracts in a controlled manner, and moves away from the incident light. This behavior allows it to navigate through a narrow channel (Figure 11H). Furthermore, fuel‐free photoactive TiO_2_ microparticles form mobile swarms under UV light via photothermal convection.^[^ ^225^ ^]^ These swarms can move and merge driven by light spots (Figure 11I). With this mechanism, long‐range migration and cargo transportation are efficiently accomplished. Exploring light‐enabled swarm control over nonphotoactive agents is also a significant area of research. One possible approach involves creating an anisotropic director field in a nematic liquid crystal suspension using light.^[^ ^226^ ^]^ Upon applying an AC electric field, pear‐shaped polystyrene microparticles propel themselves along the light‐established director field, forming a mobile swarm. The swarm can be further navigated by relocating the light spot, leveraging the light‐induced reconfiguration of the director field (Figure 11J).

### 5.2 Toward Swarm Autonomy

Swarm autonomy can be achieved through two approaches, integrating external AI and control algorithms, and designing swarms with on‐board intelligence. Artificial intelligence and on‐board intelligence are all considered as forms of machine intelligence. In this section, we investigate how swarm autonomy is realized.

#### 5.2.1 Artificial Intelligence

The integration of automatic control algorithms as external programmers has significantly advanced the precision and capabilities of microrobotic swarms, empowering them to undertake complex tasks beyond human capabilities. Automatic control is predominantly achieved in magnetic swarms due to their well‐defined kinematics model, and the real‐time programmability of magnetic actuation systems.^[^ ^219^, ^233^ ^]^ In this section, we provide an overview of the automatic control of magnetic swarms, demonstrating tasks that can be accomplished by these swarms under the guidance of automatic control algorithms. Finally, we discuss the realization of swarm autonomy through the integration of artificial intelligence.

**Figure 12:**
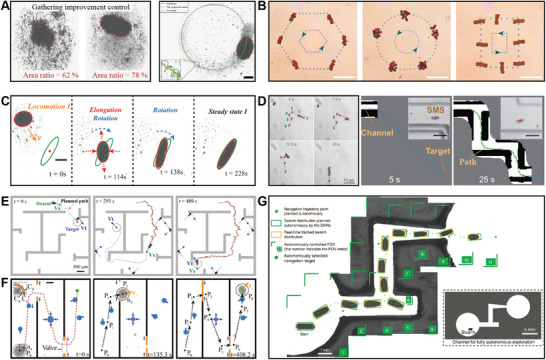
Automatic control of microrobotic swarms. A) Active generation and path following of a magnetic swarm. Reproduced with permission.^[^ ^228^ ^]^ Copyright 2020, IEEE. B) Trajectories following of magnetic swarms. Reproduced with permission.^[^ ^126^ ^]^ Copyright 2019, American Association for the Advancement of Science. C) Motion control and configuration control of a magnetic swarm. Reproduced with permission.^[^ ^206^ ^]^ Copyright 2022, IEEE. D) Automated navigation of a magnetic swarm in an unknown environment. Reproduced with permission.^[^ ^229^ ^]^ Copyright 2019, IEEE. E) Mobile target tracking of a magnetic swarm in a virtual maze. Reproduced with permission.^[^ ^230^ ^]^ Copyright 2023, IEEE. F) Dynamic obstacle avoidance of a magnetic swarm in a virtual maze. Reproduced with permission.^[^ ^231^ ^]^ Copyright 2023, IEEE. G) Autonomous navigation of a magnetic swarm enabled by a deep‐learning‐based algorithm. Reproduced with permission.^[^ ^232^ ^]^ Copyright 2022, Springer Nature.

In the field of automatic control over microrobotic swarms, there are several pivotal topics: controlled swarm generation, path following, morphology control, swarm navigation in unknown environments and dynamic environments, and swarm autonomy. **Figure** **12**A shows the controlled generation of a vortex‐like magnetic swarm enabled by a statistics‐based algorithm. Meanwhile, a trajectory tracking algorithm is implemented to guide the vortex‐like swarm, enabling it to follow a predefined circular path.^[^ ^228^ ^]^ Employing closed‐loop control strategies, magnetic swarms with distinct configurations can be automatically controlled to move along preplanned trajectories (Figure 12B).^[^ ^126^ ^]^ Furthermore, the configuration of magnetic swarms can also be automatically controlled. Using a fuzzy logical control algorithm, an elliptical magnetic swarm can be precisely guided to a specific position, and subsequently adjusted to attain a desired configuration with a different orientation and aspect ratio (Figure 12C).^[^ ^206^ ^]^

After addressing critical issues related to the automatic control of swarm locomotion and reconfiguration, new research endeavors have begun focusing on adaptive swarm locomotion within complex environments. Figure 12D demonstrates the generation and navigation of a magnetic swarm in an unknown environment.^[^ ^229^ ^]^ The generation of a snake‐like swarm is accomplished by using a Genetic algorithm. Subsequently, in this unknown environment, the swarm successfully navigates through a maze via a dynamically constructed map and a path‐planning algorithm and eventually reaches the predefined target (Figure 12D).

In the pursuit of navigating magnetic swarms in dynamic environments, significant advancements have been made by integrating automatic motion control with dynamic path planning. By employing a random tree based path planning algorithm and a genetic algorithm derived motion controller, a ribbon‐like magnetic swarm can be directed to track a mobile target within a maze with high efficiency and accuracy.^[^ ^230^ ^]^ The automatic control algorithm continuously adjusts the velocity and planned path of the magnetic swarm in real‐time based on the positions of the swarm and the moving target, while also considering the presence of obstacles (Figure 12E). Furthermore, when facing environments with dynamic obstacles, an obstacle avoidance strategy has been proposed for guiding a vortex‐like swarm through a maze.^[^ ^231^ ^]^ The proposed control algorithm is capable of handling multiple types of obstacles, including bouncing circles, expansion‐contraction circles, and open‐close gates, eventually guiding the swarm to the target (Figure 12F).

The previously mentioned automatic control of robotic swarms relies on human‐designed trajectories, parameters, controllers, and targets. However, designing machine learning algorithms to control synthetic active agents could further facilitate intelligent navigation and exploration.^[^ ^234^, ^235^ ^]^ Various computational studies have explored the incorporation of machine learning algorithms into the control of synthetic active agents.^[^ ^236^, ^237^, ^238^, ^239^, ^240^, ^241^ ^]^ The reinforcement learning algorithm has been used to train a microswimmer to navigate through a complex motility field in simulation.^[^ ^241^ ^]^ A model‐free deep neural network architecture has been developed to navigate colloidal robots in simulation, mimicking animal navigation decision‐making.^[^ ^236^ ^]^ The robot after training can make decisions based on local information to avoid obstacles and minimize traveling time. Beyond computational studies, an experimental study that incorporates reinforcement learning algorithms into the control of thermophoretic microswimmers has been conducted.^[^ ^242^ ^]^ The microswimmer is navigated in the real world with noise inputs from thermal fluctuations, hydrodynamics, and steric interactions.

Recently, through a deep learning based real‐time distribution planning algorithm, fully autonomous swarm navigation is also achieved.^[^ ^232^ ^]^ Autonomous swarm navigation includes real‐time decision‐making for position control, orientation control, pattern control, path planning, and target selection. With the autonomous algorithm, a magnetic swarm is appropriately navigated to the previously unexplored destination (Figure 12G). This work demonstrates the potential to incorporate artificial intelligence into microrobotic swarms by deploying a set of sensors, processors, and actuators outside the swarms. The intelligence of this system primarily stems from the deep learning based algorithm, which makes decisions for the entire system. It is anticipated that this approach, which incorporates machine intelligence into swarm control, holds great promise for achieving swarm autonomy.

#### 5.2.2 On‐Board Intelligence

For a formed synthetic swarm, it can be functionalized to own the capabilities of sensing physical and chemical signals from their surroundings and subsequently performing autonomous navigation and task execution.^[^ ^245^ ^]^ The emerged autonomy during this process is termed as on‐board intelligence in this review.

**Figure 13:**
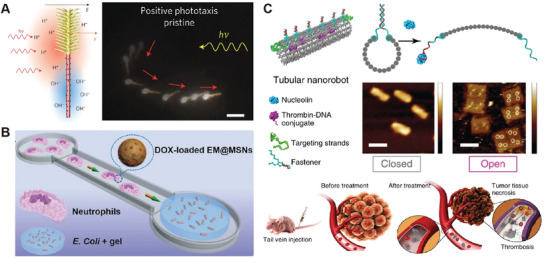
Swarm autonomy enabled by on‐board intelligence. A) Phototactic microswimmer moves toward the light source. Reproduced with permission.^[^ ^38^ ^]^ Copyright 2016, Springer Nature. B) Chemotactic biohybrid active agents move along the chemoattractant produced by *E. Coli*. Reproduced with permission.^[^ ^243^ ^]^ Copyright 2017, Wiley‐VCH. C) Autonomous DNA‐based nanorobots function as embolic agents in response to molecular triggers. Reproduced with permission.^[^ ^244^ ^]^ Copyright 2018, Springer Nature.

Autonomous navigation of synthetic swarms relies on their tactic response, which refers to the behavior of synthetic swarms to move along physical or chemical gradients. The phototactic Janus TiO_2_/Si microswimmer has been developed (**Figure** **13**A).^[^ ^38^ ^]^ The microswimmers can perform phototaxis through self‐propulsion and alignment, resulting from the self‐generated electric field under UV illumination. Based on chemically driven micromotors, chemotactic swarms have also been developed. The swarm composed of zwitterion‐based nanomotors has been developed for universal chemotactic drug delivery targeting inflammatory diseases, where ROS and iNOS serve as chemoattractants.^[^ ^246^ ^]^ Moreover, enzyme‐functionalized PLGA micromotors have been developed for chemotactic drug delivery.^[^ ^247^ ^]^ The asymmetrically functionalized catalytic enzyme can provide propulsion for the micromotors in the presence of hydrogen peroxide and drive them toward the high‐concentration region. Integrating motile microorganisms with tactic response with synthetic components provides a straightforward approach to endowing swarms with on‐board intelligence. Biohybrid micromotors composed of neutrophils and mesoporous silica nanoparticles have been developed that exhibit chemotactic response.^[^ ^243^ ^]^ These biohybrid active agents can efficiently move along the chemoattractant produced by *E. coli* (Figure 13B). Biohybrid active agents with chemotactic response have demonstrated great potential in various applications, ranging from targeted delivery to tumor penetration.^[^ ^75^, ^248^, ^249^ ^]^

Constructing stimuli‐triggered active agents with stimuli‐responsive materials is another way to achieve swarm on‐board intelligence. Stimuli‐triggered active agents can be programmed to sense environmental stimuli and respond by changing their properties for specific tasks. DNA nanorobots functioning as embolic agents in response to nucleolin, which is the protein expressed on tumor sites, have been developed (Figure 13C). These DNA nanorobots are constructed from DNA origami and locked with DNA aptamers that bind to nucleolin. Upon reaching tumor sites, the aptamers bind nucleolin and unlock the nanorobots to relaxed states, resulting in the exposure of encapsulated thrombins, which induce thrombosis to suppress tumor growth.^[^ ^244^ ^]^ Stimuli‐triggered agents that can change their motility in response to environmental stimuli have also been developed for drug delivery and cancer therapy.^[^ ^250^, ^251^, ^252^ ^]^ Urease‐powered nanorobots have been reported to exhibit enhanced diffusion and mixing capabilities in urine.^[^ ^253^ ^]^ Through intravesical administration, these nanorobots demonstrate enhanced accumulation at the tumor site of bladder cancer. Autonomous metal–organic framework nanorobots targeting mitochondria have been developed.^[^ ^254^ ^]^ These nanorobots can be energized by the hydrogen peroxide inside tumor cells to perform intracellular propulsion and cause damage to mitochondria.

### 5.3 Emergent Machine Intelligence of Synthetic Swarms

**Figure 14:**
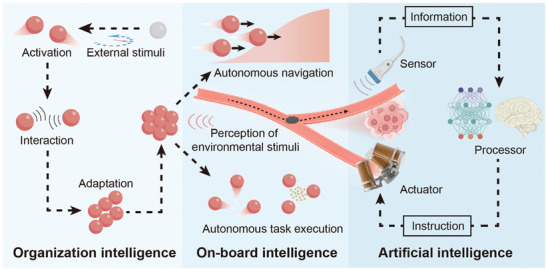
Emergent swarm autonomy with machine intelligence.

In the context of this review, we consider the machine intelligence of synthetic swarms as their ability to perceive and process information from their surrounding environment and execute specific tasks autonomously without receiving instructions from human. Our review investigates three aspects of swarm machine intelligence, i.e., organization intelligence, on‐board intelligence, and artificial intelligence (**Figure** **14**).

Organization intelligence indicates the autonomy of synthetic swarms emerged during the self‐organizing process, where the simple interaction among active agents played an important role. Fundamental processes including activation, interaction, and adaptation are involved in organization intelligence. Active agents first gain motility in response to external stimuli, such as the energization of Quincke rollers in the electric field and the propulsion of catalytic active agents in chemical fuels.^[^ ^4^, ^12^ ^]^ Subsequently, these self‐propelling agents interact with their peers via interactions, such as electrostatic, magnetic, chemical, and phoretic interaction. These interactions further lead to velocity changes of active agents, i.e., adaptation. For example, self‐propelling Janus particles energized by an electric field align their velocities upon binary collision due to electrostatic interaction.^[^ ^23^ ^]^ Such adaptations occurring at the scale of individual agents can collectively give rise to the self‐organized swarm behaviors, revealing organization intelligence.

On‐board intelligence provides swarms with the capabilities of performing programmed tasks by perceiving and responding to the surrounding environmental signals. Swarms exhibiting chemotactic response can autonomously move along chemical gradients in fluid, enabling their targeted navigation toward specific chemical signals, such as excess ROS and iNOS in inflammatory lesions.^[^ ^246^ ^]^ Additionally, stimuli‐triggered swarms can change their motilities and perform preset missions in response to the occurrence of specific environmental triggers, which enables targeted drug release, targeted embolization and tissue penetration.^[^ ^244^, ^253^, ^254^ ^]^

The synthetic swarm can also be intellectualized by integrating off‐board artificial intelligence through the incorporation of off‐board sensors, processors, and actuators. In such a robotic system, machine intelligence extends beyond the synthetic swarm and applies to the entire system. External imaging tools serve as sensors, collecting and transmitting information, while computers or humans function as central processors, processing information and making decisions. Actuation systems serve as actuators, guiding the microrobotic swarms. With the assistance of artificial intelligence, swarm autonomy can be realized through the coordination of sensors, processors, and actuators. The intelligence demonstrated by such robotic systems fundamentally reflects the artificial intelligence facilitated by computational power.

## 6 Applications Enabled by Synthetic Swarms

The diverse swarm behaviors exhibited by groups of active agents, ranging from self‐assembly to controlled locomotion, open up possibilities across various applications, including materials synthesis, device fabrication, pattern display, micro‐nano manipulation, water purification, and biomedicine.

### 6.1 Materials Synthesis

**Figure 15:**
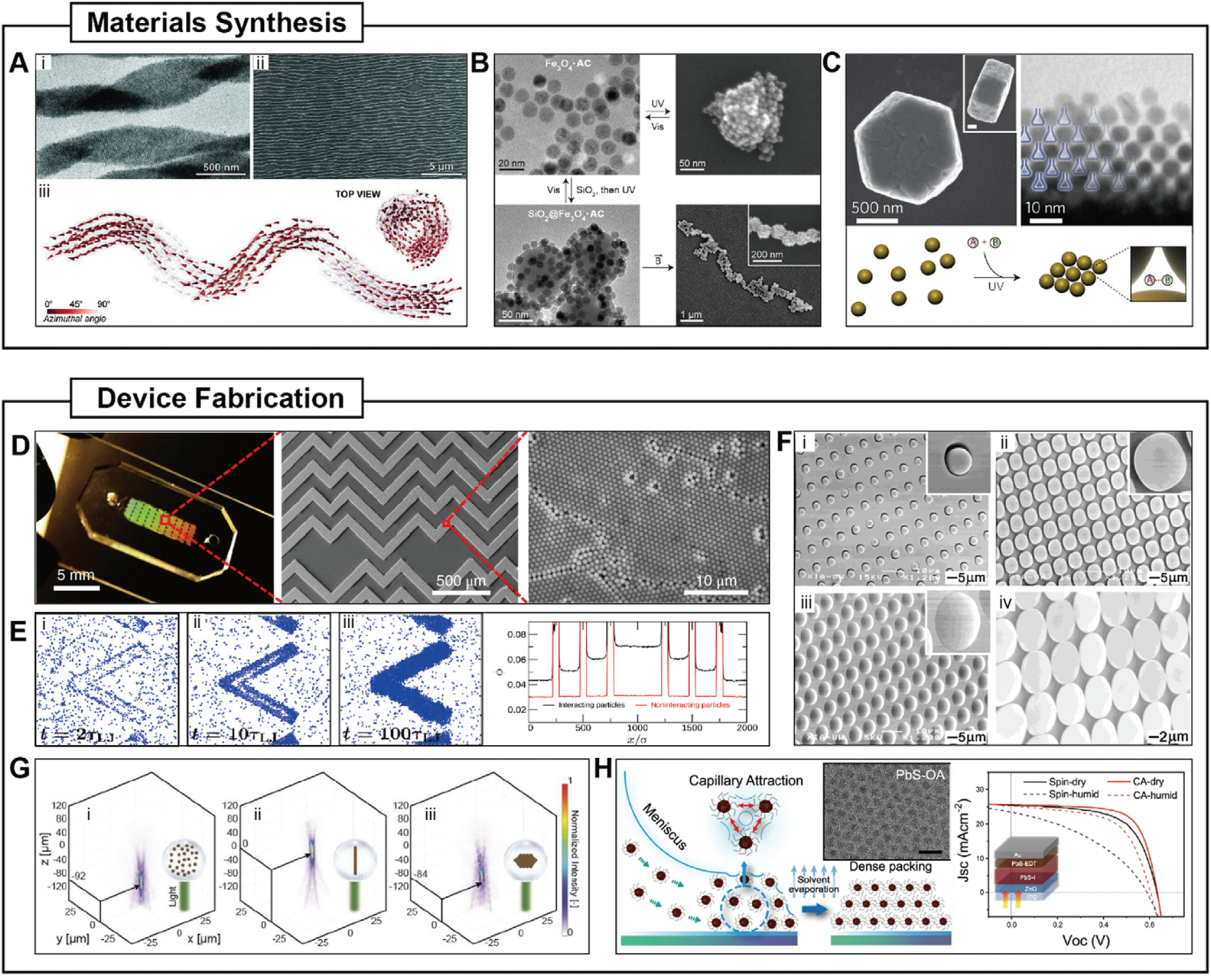
Applications of synthetic swarms in material synthesis and device fabrication. A) Synthesis of helical superstructures from magnetic nanocubes with the assistance of an external magnetic field. Reproduced with permission.^[^ ^255^ ^]^ Copyright 2014, American Association for the Advancement of Science. B) Self‐assembly of SiO_2_ nanoparticles bridged with magnetic nanoparticles under light and magnetic field. Reproduced with permission.^[^ ^256^ ^]^ Copyright 2012, American Chemical Society. C) Self‐assembled nanoreactors for chemical reactions. Reproduced with permission.^[^ ^257^ ^]^ Copyright 2016, Springer Nature. D) Microfluidic chips with nanopatterns fabricated from the self‐assembly of nanoparticles and nanorods. Reproduced with permission.^[^ ^258^ ^]^ Copyright 2019, Springer Nature. E) Proof of concept verification of a rectification device composed of light‐activated particles. Reproduced under the terms of the Creative Commons Attribution‐NonCommercial License.^[^ ^259^ ^]^ Copyright 2016, The authors, published by Association for the Advancement of Science. F) Arrays of microlenses fabricated through the self‐assembly of polystyrene microspheres. Reproduced with permission.^[^ ^260^ ^]^ Copyright 2001, Wiley‐VCH. G) Dynamic optical device composed of magnetic colloidal particles encapsulated within oil droplets. Reproduced with permission.^[^ ^261^ ^]^ Copyright 2023, Wiley‐VCH. H) Fabrication of solar cells with self‐assembled quantum dots. Reproduced under the terms of the Creative Commons Attribution 4.0 International License (http://creativecommons.org/licenses/by/4.0).^[^ ^262^ ^]^ Copyright 2021, The authors, published by Springer Nature.

Swarm behaviors, particularly self‐assembly, offer innovative approaches for fabricating superstructures of nanoparticles without the need for preexisting templates. The programmed creation of complex superstructures can be achieved by leveraging the interactions among active agents and their response to external guidance. The combination of distinct interactions, whether short‐range or long‐range, results in versatile superstructures. For instance, arrays of helical superstructures can be synthesized by leveraging the evaporation‐induced self‐assembly of magnetic nanocubes under a static external magnetic field (**Figure** **15**A).^[^ ^255^ ^]^ The formation of helical superstructures is enabled by the interplay between van der Waals and magnetic dipole–dipole interactions. It is anticipated that more complex superstructures can be fabricated through innovations in three aspects: selecting active agents with different shapes and compositions, decorating active agents with functional linkers, and implementing complex external stimuli, such as a dynamic magnetic field, to tailor the interparticle interactions. By combining light and magnetic field, the fabrication of thin films from nonmagnetic particles is realized.^[^ ^256^ ^]^ In this scenario, hydrophilic SiO_2_ nanoparticles act as building blocks, and light‐switchable azobenzene‐modified iron oxide nanoparticles serve as reversible linkers. When exposed to UV light, the *trans*‐azobenzene molecules transform into *cis*‐azobenzene, leading to the adsorption of polar azobenzene‐modified iron oxide nanoparticles on SiO_2_ nanoparticles. The subsequently applied external magnetic field induces the self‐assembly of SiO_2_ nanoparticles through magnetic attraction (Figure 15B). Self‐assembled structures can also contribute to materials synthesis by acting as nanosize reactors. For instance, colloidal nanocrystals functionalized with light‐responsive ligands can self‐assemble into a porous structure and trap various molecules from the bulk solution (Figure 15C). Within nanoconfinement, chemical reactions between the trapped molecules exhibit reaction rates and stereoselectivities that differ substantially from those in bulk solutions, providing a new approach for materials synthesis.^[^ ^257^ ^]^

### 6.2 Device Fabrication

Swarm behaviors, such as self‐assembly and shape reconfiguration, significantly contribute to the facile fabrication of various devices, ranging from microfluidic chips to semiconductor devices. For example, a 3D‐nanopatterned microfluidic chip can be fabricated through the evaporation‐induced colloidal self‐assembly of silica nanoparticles (Figure 15D).^[^ ^258^ ^]^ Following stabilization, functionalization, and encapsulation, the device can be applied for ultrasensitive detection of biomarkers like circulating exosomes. A proof‐of‐concept study verifies that light‐induced self‐assembly can be utilized for fabricating rectification devices.^[^ ^259^ ^]^ The rectification device comprises chevron‐shaped patterns assembled from light controlled particles (Figure 15E). By adjusting the intensity and spatial distribution of light, the device can be real‐time reprogrammed into distinct structures that function differently. Geometry‐guided self‐assembly enables the fabrication of optical devices, such as microlens arrays from polystyrene beads.^[^ ^260^ ^]^ During assembly, polystyrene particles are infused into a packing cell composed of two glass slides, with one of them patterned with arrays of vacancies. As the suspension flows through the cell, polystyrene beads are trapped in the vacancies. Subsequent melting turns arrays of PS beads into microlenses (Figure 15F). Furthermore, a programmable optical device can be realized by the controlled dynamic self‐assembly of magnetic nanoparticles.^[^ ^261^ ^]^ The optical device fundamentally comprises water‐in‐oil emulsions containing superparamagnetic colloids. Changing the external magnetic field leads to the formation of 2D particle assemblies with controlled configurations. These reconfigurable magnetic swarms act as reflectors that modulate the spatial distribution of light passing through them (Figure 15G). Moreover, colloidal self‐assembly is widely accepted for depositing thin films of quantum dots in devices like solar cells and LEDs.^[^ ^262^, ^263^ ^]^ Figure 15H illustrates the fabrication of quantum dots thin film for solar cells through meniscus‐guided self‐assembly. Compared to the thin film fabricated from spin coating, the self‐assembled thin film exhibits fewer vacancies, significantly reducing the self‐draining effect and promoting solar cell efficiency.^[^ ^262^ ^]^

### 6.3 Pattern Display

**Figure 16:**
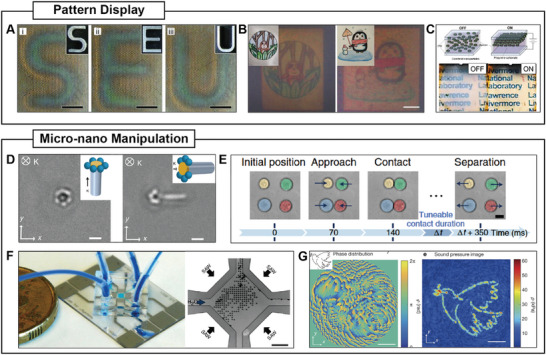
Applications of synthetic swarms in pattern display and micro‐nano manipulation. A) Patterned color display achieved by arrays of magnetic particle encapsulated microcapsules under a magnetic template. Reproduced with permission.^[^ ^264^ ^]^ Copyright 2011, Wiley‐VCH. B) Pattern display achieved by photoactive colloidal particles subjected to projected color image. Reproduced under the terms of the Creative Commons Attribution 4.0 International License (http://creativecommons.org/licenses/by/4.0).^[^ ^119^ ^]^ Copyright 2023, The authors, published by Springer Nature. C) Transparency tunable display controlled by electric field induced rearrangement of colloidal particles. Reproduced with permission.^[^ ^265^ ^]^ Copyright 2018, American Chemical Society. D) Precise manipulation of colloidal particles enabled by an optical field controlled SiO_2_ nanorod with silver cap. Reproduced under the terms of the Creative Commons Attribution 4.0 International License (http://creativecommons.org/licenses/by/4.0).^[^ ^266^ ^]^ Copyright 2019, The authors, published by Springer Nature. E) Controlled cell pairing and separation enabled by a dynamic acoustic field. Reproduced with permission.^[^ ^150^ ^]^ Copyright 2022, Springer Nature. F) Cell patterning achieved by surface acoustic waves in a microfluidic chip. Reproduced under the terms of the Creative Commons Attribution 4.0 International License (http://creativecommons.org/licenses/by/4.0).^[^ ^267^ ^]^ Copyright 2015, The authors, published by Springer Nature. G) Acoustic field induced patterning of colloidal particle assisted by hologram. Reproduced with permission.^[^ ^268^ ^]^ Copyright 2016, Springer Nature.

Reconfiguration of active agents within synthetic swarms enables their application in the field of pattern display. **Figure** **16**A shows the pattern display achieved with a magnetochromatic microcapsule array.^[^ ^264^ ^]^ These microcapsules are composed of resin shells and aqueous droplet cores filled with magnetic nanoparticles. In the absence of external magnetic fields, the magnetic nanoparticles remain suspended due to electrostatic repulsion. Upon applying an external magnetic field, the magnetic nanoparticles form ordered structures, resulting in the emergence of structural color. By using magnetic templates with specific patterns, regions subjected to the strong magnetic field exhibit structural color, thereby achieving pattern display. Through photoactivated phase segregation, photochromism enabled pattern display is achieved.^[^ ^119^ ^]^ In this scenario, photoactive colloidal particles sensitized with spectral‐distinctive dyes act as dynamic pixels. By tailoring the wavelength‐dependent interparticle pair potentials, light‐induced phase segregation of photoactive colloidal particles occurs when subjected to a projected color image (Figure 16B). Since the display originated from the redistribution of photoactive colloids with different colors, the pattern can be easily erased and reconfigured with light. Moreover, an electro‐optical device with tunable transparency is developed.^[^ ^265^ ^]^ The transparency of the device can be manipulated via electric field induced colloidal rearrangement (Figure 16C). The light reflection through the particle arrangement also contributes to the emergence of structural light, offering applications in display with tunable transparency and structural color.

### 6.4 Micro–Nano Manipulation

Active agents and their collectives can be precisely manipulated with various programmed external stimuli, including magnetic field,^[^ ^269^, ^270^, ^271^ ^]^ electric field,^[^ ^73^, ^272^ ^]^ optical field,^[^ ^137^, ^162^ ^]^ and acoustic field.^[^ ^52^, ^58^ ^]^ Micro–nano manipulation from the individual level to the collective level, from the nanometer scale to the millimeter scale can be achieved. To achieve manipulation of nano agents, a “tweezer in a tweezer” strategy is developed by combining optical tweezer and plasmonic tweezer.^[^ ^266^ ^]^ In this strategy, SiO_2_ nanorods with silver caps act as plasmonic tweezers that can trap nanoscale colloidal nanoparticles. The nanorods can be further manipulated by an external optical field for long‐range transportation (Figure 16D). The acoustic field shows huge potential in manipulating colloidal particles and biological cells for their versatility and bio‐compatibility. With interdigital transducers (IDTs), surface acoustic waves with tunable frequency and amplitude can be generated, which leads to the creation of an acoustic field with tunable trapping positions.^[^ ^150^ ^]^ Through manipulating the trapping positions, colloidal particles, or cells can be reversibly assembled and separated (Figure 16E). Combining with a microfluidic chip, a large number of cells can be patterned into 2D arrays within the acoustic field, offering opportunities in biological cell analysis and life science research (Figure 16F).^[^ ^267^ ^]^

Holographic techniques, combined with acoustic fields, enable controlled assembly of colloidal particles into large‐scale desired patterns.^[^ ^268^ ^]^ Holographic techniques enable the spatial storage of the phase and amplitude profile of the desired wavefront. When a propagating wave with a coherent source passes through the hologram, its wavefront is reconstructed by interference. Combining the hologram with the acoustic field, sound pressure with the desired pattern can be created and enable large‐scale manipulation (Figure 16G). These advanced manipulation techniques, using optical, plasmonic, and acoustic stimuli, open new possibilities in the precise control of micro‐nanoscale entities.

### 6.5 Water Purification

**Figure 17:**
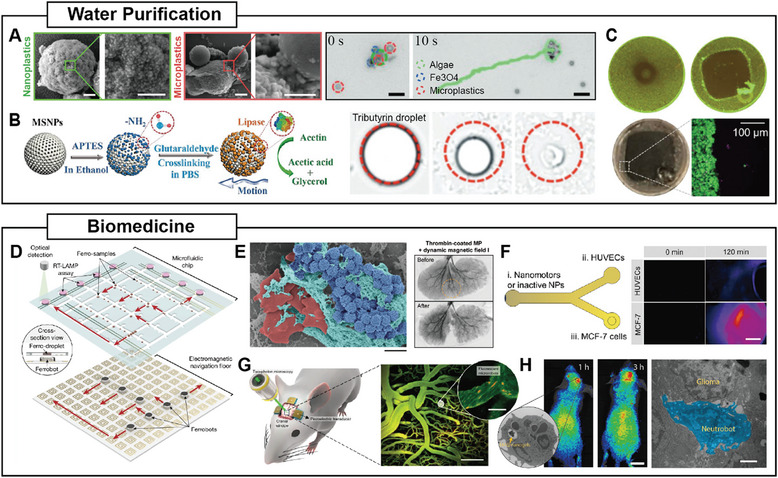
Applications of synthetic swarms in water purification and biomedicine. A) Magnetic algae robots for plastic removal. Reproduced under the terms of the Creative Commons CC BY license.^[^ ^273^ ^]^ Copyright 2023, The authors, published by Wiley‐VCH. B) Chemical fuel energized micromotors capable of removing triglyceride. Reproduced with permission.^[^ ^274^ ^]^ Copyright 2019, Wiley‐VCH. C) Cluster of magnetic particles modified with biofilm debris for biofilm eradication. Reproduced with permission.^[^ ^147^ ^]^ Copyright 2019, Association for the Advancement of Science. D) Viral testing enabled by a swarm of individually addressable magnets. Reproduced with permission.^[^ ^275^ ^]^ Copyright 2022, Springer Nature. E) Selective embolization achieved by magnetic particles modified with thrombin. Reproduced under the terms of the Creative Commons Attribution License 4.0 (https://creativecommons.org/licenses/by/4.0).^[^ ^276^ ^]^ Copyright 2022, The authors, published by Association for the Advancement of Science. F) Chemotactic synthetic polymer nanoparticles migrate toward cancer cells. Reproduced with permission.^[^ ^246^ ^]^ Copyright 2022, Wiley‐VCH. G) Two‐photon microscopic imaging of mouse brain vascular with ultrasound‐actuated microbubbles. Reproduced under the terms of the Creative Commons Attribution 4.0 International License (http://creativecommons.org/licenses/by/4.0).^[^ ^149^ ^]^ Copyright 2023, The authors, published by Springer Nature. H) Magnetic nanogel encapsulated neutrophils for targeted delivery in the brain. Reproduced with permission.^[^ ^75^ ^]^ Copyright 2021, Association for the Advancement of Science.

Self‐propelled active agents offer a promising solution for water remediation by enhancing interactions with pollutants and overcoming the limitations of diffusion‐limited chemical reactions. Water pollutants, including microplastics,^[^ ^273^, ^277^ ^]^ organic pollutants,^[^ ^278^ ^]^ heavy metals,^[^ ^279^ ^]^ and microorganisms,^[^ ^147^ ^]^ have been verified can be effectively removed with self‐propelled active agents. For instance, algae‐based microrobots can be created by coating Fe_3_O_4_ nanoparticles on algae cells to remove plastics.^[^ ^273^ ^]^ These magnetic algae robots can be steered by the external magnetic field and capture plastics through electrostatic attraction (**Figure** **17**A). The attractive phoretic interaction between active agents and passive pollutants provides an alternative approach for efficient plastic removal. For example, TiO_2_‐based micromotors can collect microplastics, such as the remnants of personal care products, by light‐induced phoretic interaction.^[^ ^277^ ^]^ Active agents also showcase capabilities of removing harmful organics existing in the human body. For instance, self‐propelled micromotors capable of removing triglycerides are developed.^[^ ^274^ ^]^ These micromotors, composed of mesoporous silica nanoparticles and lipase coatings, propel themselves through biocatalytic reactions, accelerating the degradation of triglyceride in solution (Figure 17B). Moreover, polypyrrole‐based microrobots integrated with Pt catalytic layer and magnetic nanoparticles have been developed for efficient removal of oestrogenic pollutants.^[^ ^278^ ^]^ Pollutants caused by microorganisms, such as biofilm can also be effectively removed by collectives of active agents. For example, biofilm eradication has been achieved by biohybrid agents composed of iron oxide nanoparticles and biofilm debris.^[^ ^147^ ^]^ These agents can be concentrated using a permanent magnet and guided to move on the biofilm with a predesigned path, to locally kill bacteria and degrade the biofilm matrix (Figure 17C).

### 6.6 Biomedicine

Active agents are revolutionizing various aspects of biomedicine, from biomarker extraction to targeted therapy.^[^ ^39^, ^280^, ^281^, ^282^, ^283^, ^284^ ^]^ For example, antibody‐modified magnetic beads can be used to extract circulating tumor cells, tumor‐reactive lymphocytes, and exosomes from blood samples.^[^ ^285^, ^286^, ^287^ ^]^ The on‐demand dispersion and aggregation of magnetic beads greatly enhance their biomarker extraction efficiency. Magnetic active agents can also be incorporated with a testing platform to achieve automated viral testing. For example, a swarm of millimeter‐sized magnets is integrated with a testing platform to handle magnetized sample droplets.^[^ ^275^ ^]^ The platform consists of a microfluidic chip for sample holding and operation and an underneath printed circuit board (PCB) with electromagnetic coils for actuation. These magnetic agents are individually addressable and can be controlled parallel to perform tasks like aliquoting, droplet merging, mixing, and heating (Figure 17D). With a programmed control scheme, the automated nucleic acid amplification tests can be accomplished by the ferrobotic swarm.

Moreover, swarms of micro‐nanoscale active agents offer the opportunity to noninvasively access hard‐to‐reach regions inside the human body, demonstrating huge potential in biomedical applications such as targeted delivery, medical imaging, and nanosurgery. For example, selective embolization has been achieved by swarms of thrombin‐modified magnetic agents driven by a rotating magnetic field with a time‐averaged local amplification.^[^ ^276^ ^]^ Magnetic agents are more likely to form aggregates in the desired region with higher magnetic field strength. With specially designed magnetic fields and thrombin‐modified magnetic particles, in vivo selective embolization on the porcine kidney is realized (Figure 17E). Leveraging the chemotaxis mechanism, active agents that spontaneously move toward sites where reactive oxygen species (ROS) and inducible nitric oxide synthase (iNOS) overexpress are developed.^[^ ^246^ ^]^ These nanomotors are composed of polymer nanoparticles and l‐arginine (l‐Arg) as catalytic doping. The l‐Arg can be catalyzed by iNOS and react with ROS in the physiological environment and produce NO, leading to self‐propulsion and chemotaxis. It is demonstrated that these nanomotors can spontaneously migrate to cancer cells in a simulated environment, verifying their potential for targeted cancer therapy (Figure 17F).

Functionalizing active agents with contrast agents enables real‐time imaging and navigation of synthetic swarms inside the human body, which significantly facilitates their biomedical applications.^[^ ^288^, ^289^ ^]^ Active agents can be engineered to be compatible with various imaging tools, including X‐ray imaging,^[^ ^290^, ^291^ ^]^ ultrasound imaging,^[^ ^127^, ^292^, ^293^, ^294^ ^]^ photoacoustic imaging,^[^ ^114^, ^295^, ^296^ ^]^ and fluorescence imaging.^[^ ^297^, ^298^, ^299^ ^]^ For example, lipid‐coated microbubbles have been used for optical imaging.^[^ ^149^ ^]^ They can be propelled by acoustic fields in the vascular system. Using the two‐photon microscopy, navigation of these agents in the mouse brain vasculature is demonstrated and the 3D cerebral capillary network is reconstructed (Figure 17G).

Combining guided locomotion and surface functionalization, active agents hold significant promise in targeted delivery. The inherent motility of active agents enables their navigation to target regions inside the human body through open channels, such as the circulatory system, gastrointestinal system, and respiratory system.^[^ ^84^, ^85^, ^114^, ^284^, ^285^, ^293^, ^300^ ^]^ The surface functionalization can either serve as camouflage for active agents to avoid immune clearance and cross physiological barriers,^[^ ^75^, ^113^, ^281^, ^301^ ^]^ or enable loading of therapeutic cargos like drugs,^[^ ^302^ ^]^ stem cells,^[^ ^303^ ^]^ and genes.^[^ ^304^, ^305^ ^]^ For example, cell‐based magnetic agents are developed by incorporating magnetic nanogels into neutrophils.^[^ ^75^ ^]^ These microrobots can be guided to the brain under a rotating magnetic field and pass through the blood–brain barrier through positive chemotaxis toward inflammatory factors, thereby delivering drugs to the malignant glioma (Figure 17H).

### 6.7 Insights on Applications of Synthetic Swarms

The functionalities and potential applications of synthetic swarms are intrinsically related to the properties of individual active agents. We classify these agents into three categories, based on their responsive properties and functionalization as inert agents, responsive agents without functionalization, and responsive agents with functionalization. Inert agents do not alter their properties under external stimuli, while the properties of responsive agents can change with external stimuli. The representative applications of synthetic swarms are summarized in **Table** **2**, considering key aspects of agent properties, swarm behaviors, external programmers, and applications. We observe that most applications are enabled by synthetic swarms composed of functionalized active agents. Further development in agent functionalization represents an important area of research, which could significantly advance practical applications of synthetic swarms. Moreover, most current applications rely on the spontaneous behaviors of swarms, often associated with static or equilibrium states of these swarms. Looking to the future, dynamic swarms, such as microrobotic swarms, driven by programmable external stimuli, present a frontier yet to be fully investigated. Real‐world applications of synthetic swarms in complex environments, like inside the human body, would require the intervention of human or artificial intelligence. This integration of intelligence could unlock a wide range of complex applications, from targeted therapeutic delivery to nanosurgical procedures. We anticipate that the evolving field of synthetic swarms, driven by advancements in agent design and intelligent control, will significantly broaden its impact in various practical applications.

**Table 2:** Representative application enabled by synthetic swarms.

Agents		Swarm behaviors	Applications	External programmer	Refs.
Inert agents	PS microsphere	Self‐assembly	Device fabrication (Micro lenses)	N.A	[260]
	PDMS microsphere	Cluster	Manipulation	N.A.	[268]
	Magnets	Swarm	Biomedicine (Viral test)	Computer	[275]
Responsive agents without functionalization	Magnetic nanocube	Self‐assembly	Nanosynthesis	N.A.	[255]
	Ag/SiO_2_ nanorod	Cluster	Manipulation	Human	[266]
	ZnS/SiO_2_ nanoparticle	Cluster	Pattern display	N.A.	[265]
Responsive agents with functionalization	Azobenzene‐modified iron oxide particle	Self‐assembly	Nanosynthesis	Human	[256]
	Light‐responsive ligands modified nanocrystal	Self‐assembly	Nanosynthesis	N.A.	[257]
	Antibody‐modified silica nanoparticle	Self‐assembly	Device fabrication (Exosomes extraction)	N.A.	[258]
	Magnetic self‐adhesive microgel	Cluster	Biomedicine (Embolization)	N.A.	[290]
	Thrombin‐modified magnetic particles	Cluster	Biomedicine (Embolization)	N.A.	[276]
	Dye‐sensitized SiO_2_	Cluster	Pattern display	N.A.	[119]
	Magnetic microcapsule	Cluster	Pattern display	N.A.	[264]
	Magnetic nanogel / Neutrophil	Swarm	Biomedicine (Targeted delivery)	N.A.	[75]
	L‐Arg coated polymer nanoparticle	Swarm	Biomedicine (Targeted delivery)	N.A.	[246]
	Biofilm debris / Iron oxide	Swarm	Water purification (Biofilm eradiation)	Human	[147]
	Lipid coated microbubbles	Swarm	Biomedicine (Medical Imaging)	Human	[149]

## 7 Summary and Outlook

This review presents a thorough overview of synthetic swarms, beginning with an analysis of the design of active agents, detailing various types of stimuli they can exploit and the range of motility and functionalities they can exhibit. A critical part of our discussion focuses on the mechanism enabling agent communication and coordination, which are instrumental in the emergence of swarm behaviors. Furthermore, we examine the field of swarm autonomy empowered by artificial intelligence, elucidating how synthetic swarms, acting as microrobots, integrate off‐board sensors, processors, and actuators to demonstrate intellectual behaviors. Finally, we bridge the features of individual agents and swarm behaviors with their practical applications, offering insights into the design of synthetic swarms.

Challenges and opportunities in the field of synthetic swarms lie in two aspects: designing active agents with intrinsic intelligence, and implementing autonomous swarms for real‐world applications. Depending on the exhibited complexity, agents or matter can be classified into four categories, inert, responsive, adaptive, and intelligent.^[^ ^306^ ^]^ Current synthetic swarms primarily comprise inert agents and responsive agents. Inert agents refer to agents that cannot change their properties after synthesis, while responsive agents change their properties in response to external stimuli. However, the stimuli‐responsive behaviors are predetermined and strictly adhere to fixed rules. Beyond responsive agents, there are adaptive agents, which not only respond to external stimuli but also regulate their internal feedback mechanism adaptively. Such modulation requires computational units capable of processing information and making decisions. Furthermore, intelligent agents are capable of learning from input information and self‐regulating their actions, which requires learning capabilities and memory functions. Incorporating adaptive or intelligent agents into synthetic swarms can fundamentally increase the complexity of the systems, thereby constructing synthetic swarms with higher levels of intelligence and capabilities.^[^ ^307^ ^]^ Nevertheless, active agents in synthetic swarms are mostly colloidal particles with sizes smaller than 100 µm, constructing adaptive and intelligent agents integrated with computational and memory units presents substantial challenges.^[^ ^308^ ^]^ Solution may emerge from two approaches, fabricating silicon‐based microsystems that integrate microelectronics for sensing, computation, communication, and actuation;^[^ ^309^ ^]^ and innovating smart materials with inherent adaptability and intelligence.^[^ ^245^ ^]^ We anticipate that increasing the complexity and intelligence of individual agents will significantly enhance the diversity and complexity of synthetic swarms.

The realization of swarm autonomy in real‐world applications presents the second challenge. Synthetic swarms, with their distinct functionalities, find applications in diverse fields, from advanced material synthesis to biomedicine. Among these applications, the application of microrobotic swarms in minimally invasive medicine is particularly fascinating and holds immense promise.^[^ ^284^ ^]^ Before deploying autonomous swarms within the human body, two critical issues must be considered. First, the integration of artificial intelligence into the automatic control of mirorobotic swarms to enhances their adaptability and intelligence. This advancement enables the microrobotic swarms to intelligently navigate in complex and dynamic physiological environments. Second, the incorporation of real‐time medical imaging techniques, such as X‐ray or ultrasound, into swarm control algorithms.^[^ ^310^ ^]^ This integration provides continuous monitoring and feedback regarding the swarm location and behavior, which is essential for precise maneuvering and task execution within the body. Combining the intrinsic functionalities of individual agents with enhanced swarm autonomy opens up new opportunities in biomedical applications, spanning from targeted delivery to advanced medical imaging. Looking ahead, we anticipate that the widespread implementation of robotic systems based on microrobotic swarms for a variety of applications could be a reality in the near future.

## Conflict of Interest

The authors declare no conflict of interest.
